# Expansion of ventral foregut is linked to changes in the enhancer landscape for organ-specific differentiation

**DOI:** 10.1038/s41556-022-01075-8

**Published:** 2023-01-23

**Authors:** Yan Fung Wong, Yatendra Kumar, Martin Proks, Jose Alejandro Romero Herrera, Michaela Mrugala Rothová, Rita S. Monteiro, Sara Pozzi, Rachel E. Jennings, Neil A. Hanley, Wendy A. Bickmore, Joshua M. Brickman

**Affiliations:** 1 Novo Nordisk Foundation Center for Stem Cell Medicine (reNEW), University of Copenhagen, Copenhagen, Denmark; 2 MRC Human Genetics Unit, Institute of Genetics and Cancer, University of Edinburgh, Edinburgh, UK; 3 Center for Health Data Science, University of Copenhagen, Copenhagen, Denmark; 4 Faculty of Biology, Medicine & Health, Manchester Academic Health Science Centre, University of Manchester, Manchester, UK

**Keywords:** Cell proliferation, Epigenomics, Stem-cell differentiation

## Abstract

Cell proliferation is fundamental for almost all stages of development and differentiation that require an increase in cell number. Although cell cycle phase has been associated with differentiation, the actual process of proliferation has not been considered as having a specific role. Here we exploit human embryonic stem cell-derived endodermal progenitors that we find are an in vitro model for the ventral foregut. These cells exhibit expansion-dependent increases in differentiation efficiency to pancreatic progenitors that are linked to organ-specific enhancer priming at the level of chromatin accessibility and the decommissioning of lineage-inappropriate enhancers. Our findings suggest that cell proliferation in embryonic development is about more than tissue expansion; it is required to ensure equilibration of gene regulatory networks allowing cells to become primed for future differentiation. Expansion of lineage-specific intermediates may therefore be an important step in achieving high-fidelity in vitro differentiation.

## Abstract (web summary)

Wong et al. report that expanding human embryonic stem cell-derived endodermal progenitors serve as a ventral foregut model, through which they identify a link between progenitor expansion and tissue-specific changes in the enhancer landscape.

## Main

The regulation of gene expression during differentiation is considered a linear process involving the action of signalling and transcription factors (TFs). Cell proliferation is regarded as peripheral to differentiation, although it has a clear function in the selection of specific cell types. While cell cycle phase has been linked to differentiation^1,2^, here we explore the notion that differentiation requires progenitor proliferation itself to enhance the processing of lineage-promoting information.

The visceral organs are formed during embryonic development from the endoderm germ layer^3^. These cells are initially specified during gastrulation and undergo extensive proliferation as they prepare to differentiate into distinct organ primordia^4^. In particular, the liver and pancreas are derived from the anterior definitive endoderm (ADE). ADE is formed as a result of the anterior migration of cells from the anterior region of the primitive streak at the beginning of gastrulation. The anterior-most definitive endoderm (DE) will then migrate ventrally to form the ventral foregut, containing a bipotent precursor of liver and ventral pancreas^5,6^, a population that has recently been shown to expand and retain potency for both lineages in vivo over a period of several days of mouse development^7^.

Pluripotent embryonic stem cells (ESCs) can be differentiated in vitro to form all embryonic germ layers including endoderm^8,9^. As a result, directed linear ESC differentiation is used to produce organ-specific cell types such as pancreatic beta cells^10–12^ and hepatocytes^13,14^. An alternative to directed differentiation is the use of ESC-derived expandable endodermal progenitors (EPs) as a staging platform for further differentiation^15–17^, and the expansion of endodermal cells from human ESCs (hESCs) promotes the generation of more mature pancreatic beta cells^15^.

In this Article, we find that the in vivo identity of human EP (hEP) cells is ventral foregut and that continued proliferation of these cells results in lineage priming that is correlated with organ-specific enhancer accessibility. Lineage priming is not accompanied by large changes in transcription of organ-specific genes, but instead prepares appropriate enhancers for their activation and decommissions enhancers normally present in other lineages. Our findings suggest that the extensive cell proliferation that characterizes normal embryonic development is not merely required for tissue expansion, but ensures equilibration of gene regulatory networks for future high-fidelity differentiation.

## Results

### Expanding endoderm progenitors mimic ventral foregut in vitro

**Fig. 1:**
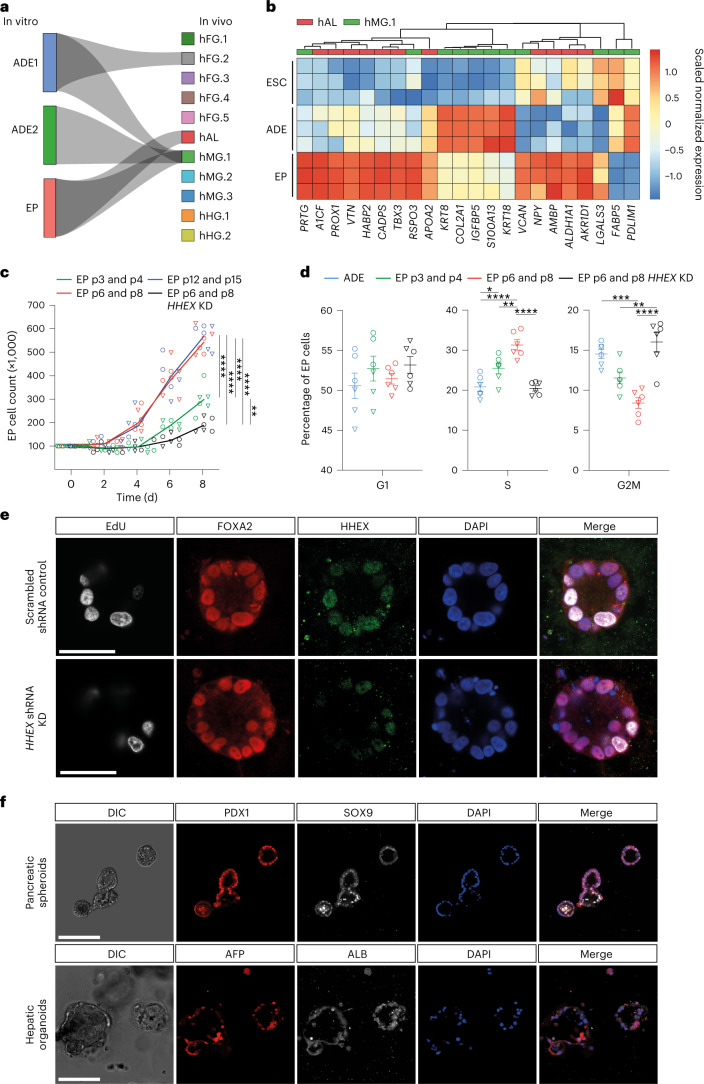
Expanding endoderm progenitors as an in vitro model for ventral foregut. **a**, Visualization of the CAT alignments between in vitro clusters (ADE.1, ADE.2 and EP) from this study and in vivo endodermal clusters from the Li et al. dataset^20^. Only significant CAT alignments between clusters are shown. **b**, Heat map showing expression of hAL and hMG marker genes in ESC, ADE and EP cells (bulk RNA-seq dataset, scaled normalized expression, *N* = 3 independent experiments). Only markers expressed significantly different between ADE and EP are shown (log_2_FC > 1.5, adjusted *P* < 0.05). **c**, Cumulative growth curves showing EP cell counts at different passages of expansion for control and HHEX KD (EPs were derived from H9 (circle) or HUES4 (triangle) ESCs). Data are represented as mean ± s.e.m.; *N* = 6 independent experiments. **P < 0.01, ****P < 0.0001 (one-way ANOVA Tukey’s multiple comparison test was applied to analyse differences at day 8; only significant comparisons are shown). **d**, Dot plots showing percentage of G1, S and G2M cycling cells assayed by flow cytometry with EdU and DAPI staining in control and HHEX KD EP expansion. Data are represented as mean ± s.e.m.;*N* = 6 independent experiments. *P < 0.05, **P < 0.01, ***P < 0.001, ****P < 0.0001 (one-way ANOVA Tukey’s multiple comparison test; only significant comparisons are shown). **e**, Representative images (from three independent experiments) of control (top row) and HHEX shRNA (bottom row) EP cells stained with EdU, FOXA2, HHEX and DAPI. Scale bars, 50 µm. **f**, Top: representative immunostaining of PDX1 and SOX9, including DAPI, of VFG-derived pancreatic spheroids at passage 5. Bottom: representative immunostaining of AFP and ALB, including DAPI, of VFG-derived hepatic organoids at passage 5. Images represent three independent experiments. Scale bars, 50 µm. DIC, differential interferance contrast. Source data

**Extended Data Fig. 1:**
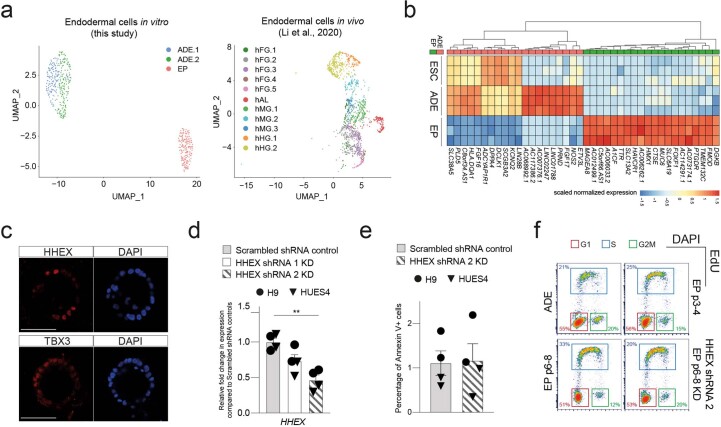
Ventral foregut identity of expanding endodermal progenitors. **a**, Left: UMAP visualization of single cells from the transient ADE (ADE.1 and ADE.2) and EP passage 6 samples. Right: UMAP visualization of single cells from different endodermal populations from early human embryos reported in Li et al.^20^. **b**, Heatmap illustrating gene expression in H9-derived ESC, ADE, and EP cells (*N* = 3 independent experiments) from bulk RNA-seq dataset. Scaled normalized expression of the top 20 differentially expressed genes for each condition (ADE vs EP) is shown. **c**, Representative immunostaining of hAL markers, HHEX and TBX3, in EP passage 6 cells derived from H9 ESC cells. Images represent three independent experiments. Scale bar = 50 µm. **d**, Expression analysis in HHEX shRNA KD cells (set 1 and set 2) and scrambled shRNA control by RT-qPCR. Relative fold change in mRNA of HHEX gene in KDs and control EP/VFG cells was assayed by RT-qPCR. Expression is normalized to *ACTB*. Circles and triangles mark cells derived from H9 and HUES4 WT ESCs respectively. Data are represented as mean ± SEM (*N* = 4 independent experiments). Statistical analysis (**P < 0.01, unpaired two-tailed t-test) was performed between KD and control EP/VFG cells. Comparisons without an indicated P value are not significant. **e**, Apoptosis assay in HHEX shRNA (set2) KD and scrambled shRNA control EP/VFG. Bar plot showing percentage of Annexin V + cells for each assay. Circles and triangles mark cells derived from H9 and HUES4 WT ESCs respectively. Data are represented as mean ± SEM (*N* = 4 independent experiments). No statistical difference (unpaired two-tailed t-test) was found between HHEX shRNA KD and scrambled shRNA control. **f**, Representative flow cytometry plots used to analyze the cell cycle in transient ADE, early EP/VFG (p3-4), expanding EP/VFG (p6-8) and HHEX depleted EP/VFG (p6-8). Cells were stained with EdU and DAPI. Cells in G1 (red), S (blue), and G2M (green) were gated and percentages of each fraction shown. Flow Cytometry plots represent three independent experiments. Source data

To characterize the impact of expansion on endodermal differentiation, we focused on 3D hEP culture^15^. This protocol expands endoderm in the presence of FGF2, BMP4, VEGF and EGF^15,17^, cytokines known to act in the ventral foregut region. We quantitated gene expression during expansion by single-cell RNA sequencing (RNA-seq) and found that transient ADE cells comprised two subpopulations (ADE.1 and ADE.2) while EP culture was homogeneous (Extended Data Fig. 1a, left). In human development, ventral foregut endoderm has been described at Carnegie stages 8 and 9 (ref. ^18^), and we compared hESC-derived endoderm with single-cell RNA-seq from these stages of human embryos with our cluster alignment tool (CAT)^19^. For this analysis, we used a recently published dataset containing human embryonic foregut (hFG.1-4), the lip formed from ventral foregut—referred to as the lip of the anterior intestinal portal (hAL)—midgut (hMG.1-3) and hindgut (hHG.1-2) (ref. ^20^) (Extended Data Fig. 1a, right). We found that ADE aligns to the foregut hFG.2 and midgut hMG1 clusters (Fig. 1a). In contrast, EP cells align with hAL and hMG1, a population of midgut cells located adjacent to the hAL^20^. As EP cells align to both these clusters, we assessed gene expression specifically enriched in RNA-seq from H9-derived EP cells (Extended Data Fig. 1b) and asked whether this set contained genes with differential expression between hAL and hMG.1 clusters. With a few exceptions, genes expressed at higher levels in hAL were also elevated in EP cells (Fig. 1b). The hAL or ventral foregut identity of EP cells was confirmed by immunohistochemistry of the hAL markers HHEX^20^ and TBX3 (ref. ^15^) (Extended Data Fig. 1c).

As murine ventral foregut endoderm is actively cycling^21^, we measured the proliferation rate of hEP cultures and found it increased with time in culture (p6, p8, p12 and p15) (Fig. 1c). In mouse, HHEX is known to support ventral foregut expansion and morphogenesis^21^. To further confirm the identity of hEP, we knocked down HHEX by short hairpin RNA (shRNA) and observed a reduction in growth without induction of apoptosis (Extended Data Fig. 1d,e). We measured actively proliferating cells in ADE, EP cells and HHEX knockdown (KD) EP cells by 5-ethynyl-2′-deoxyuridine (EdU) labelling followed by cell-cycle analysis based on 4′,6-diamidino-2-phenylindole (DAPI) staining (Extended Data Fig. 1f). The percentage of S-phase cells increased with expansion in an HHEX-dependent fashion, while the fraction in G2M was reduced (Fig. 1d,e). On the basis of the expression of ventral foregut markers, the cytokines used in these cultures and the function of HHEX in proliferation, we conclude hEP cells are an in vitro model for human ventral foregut and refer to them hereafter as ventral foregut progenitor cells (VFGs).

**Extended Data Fig. 2:**
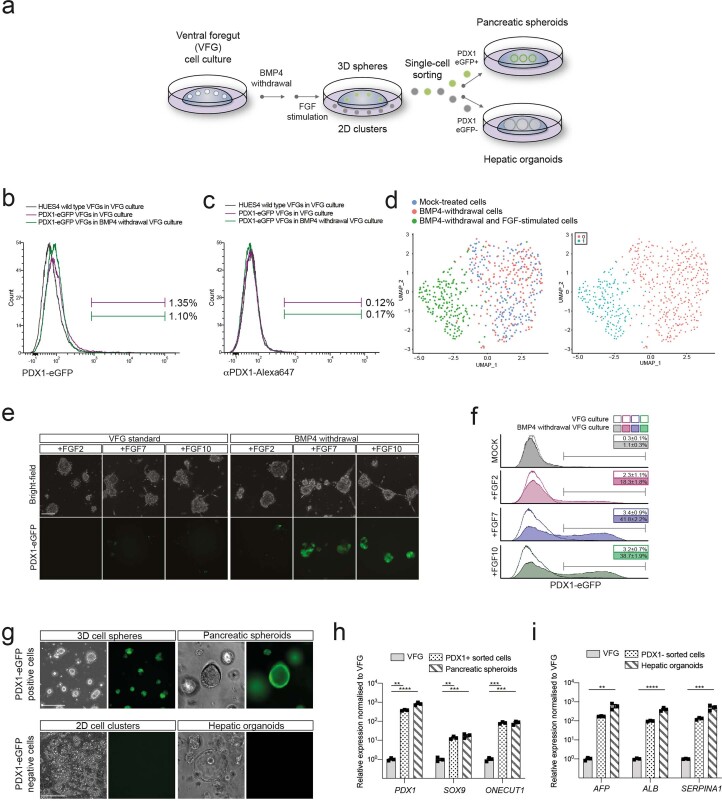
Human VFG cultures can be readily transformed to either pancreatic or hepatic lineages. **a**, Schematic representation showing the conversion of VFG culture to pancreatic and hepatic expansion. The figure illustrates the generation of PDX1^−^eGFP positive (PDX1^+^) and negative (PDX1^−^) cells from VFG culture after BMP4 withdrawal and subsequent stimulation with FGF. **b-c**, Flow cytometry of eGFP expression (**b**) or intracellular PDX1 (**c**) for HUES4 wild type (grey), PDX1^−^eGFP reporter (purple) in VFG culture, and the reporter following BMP4 withdrawal (green). Fractions of eGFP^+^ or PDX1^+^ were gated and percentages are shown. Flow Cytometry plot represents three independent experiments. **d**, Left: UMAP visualization of 526 cells isolated from mock-treated VFG (blue), VFG cells grown in the absence of BMP4 (red), and transient pancreatic induction by FGF2 simulation (green). Right: UMAP visualization of Seurat clustering from the samples described on the left. **e**, Representative bright-field (top) and fluorescent (bottom) images for the PDX1-eGFP reporter VFGs (left) or following BMP4 withdrawal (right), and then treated with FGF2, FGF7, or FGF10. Images represent three independent experiments. Scale bar = 50 μm. **f**, Flow cytometry of eGFP expression for the conditions described in (**e**), including mock-treated cells. Percentages of PDX1^+^ cells were shown in the rectangle boxes of each histogram. Flow Cytometry plots represent three independent experiments. **g**, PDX1^+^ cells form 3D spheres and expand as pancreatic spheroids (Top). PDX1^−^cells form 2D clusters and expand as hepatic organoids (bottom). Images represent three independent experiments. Scale bar = 50 µm. **h-i**, Relative fold change in mRNA of pancreatic markers (*PDX1*, *SOX9*, and *ONTCUT1*) (**h**) and hepatic markers (*AFP*, *ALB*, and *SERPINA1*) (**i**) in the VFGs and VFG-derived cell types (as described in **a** and **g**). Expression is normalized with *ACTB*. Data are represented as mean ± SEM (*N* = 3 independent experiments). **P < 0.01, ***P < 0.001, ****P < 0.0001 (one-way ANOVA Dunnett’s multiple comparison test compared with VFG). Source data

To probe VFG differentiation efficiency, we established VFG cultures from an hESC line containing a pancreatic reporter (PDX1-eGFP)^22^ and determined the minimal cytokine set required to transform VFG spheres into proliferating pancreatic spheroids or hepatic organoids (Extended Data Fig. 2a). Removal of BMP4 from VFG culture resulted in negligible PDX1 reporter expression (<2% GFP^+^), no PDX1 protein and no dramatic transcriptional change at single-cell level (Extended Data Fig. 2b–d). Subsequent addition of FGF7 and FGF10, and to a lesser extent FGF2, stimulated PDX1-eGFP expression and induced robust transcriptional change (Extended Data Fig. 2d–f). In response to initial cytokine treatment, we could separate PDX1^+^ and PDX1^−^ cells, and expand PDX1^+^ cells as pancreatic spheroids, or PDX1^−^ cells as hepatic organoids (Fig. 1f and Extended Data Fig. 2g–i) in defined media^23,24^. These observations indicate that human VFG culture is poised to generate expanding hepatic and pancreatic endoderm.

### Expansion enhances pancreatic differentiation of VFG cells

**Fig. 2:**
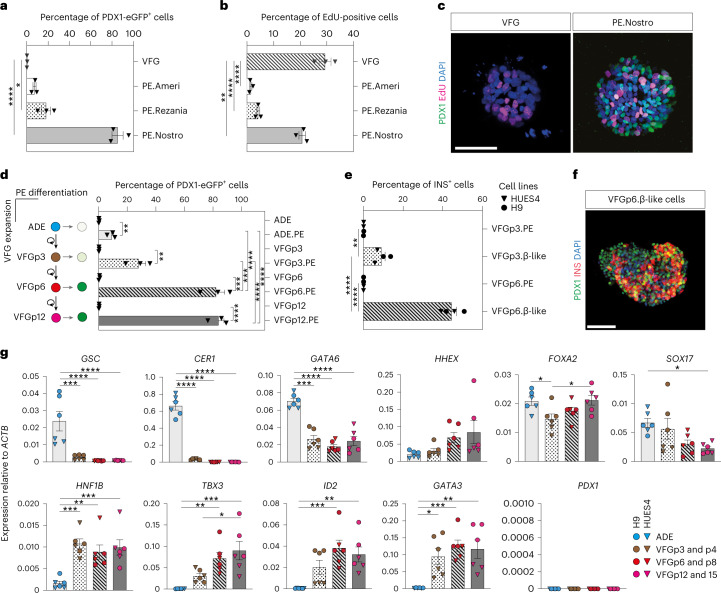
Expansion enhances pancreatic differentiation of VFG cells. **a**,**b**, Bar plots showing percentage of PDX1-eGFP^+^ (**a**) or EdU^+^ (**b**) cells from flow cytometry analysis in VFG cells and PE generated from VFG cells on the basis of different differentiation protocols. Data are represented as mean ± s.e.m.; *N* = 3 independent experiments. *P < 0.05, ****P < 0.0001 (one-way ANOVA Dunnett’s multiple comparison test compared with VFG cells). **c**, Representative immunostaining (from three independent experiments) of VFG cells and PE generated using conditions from Nostro et al. (ref. ^10^), stained with PDX1, EdU and DAPI. Scale bar, 50 µm. **d**, Left: schematic of PE differentiation using conditions from Nostro et al. (ref. ^10^). from ADE and VFG at p3, p6 and p12. Right: bar plot showing percentage GFP^+^-positive cells generated for the indicated conditions. Data are represented as mean ± s.e.m.; *N* = 3 independent experiments. Statistical analysis was performed for differentiation of each indicated cell type (**P < 0.01, ***P < 0.001, ****P < 0.0001, unpaired two-tailed *t*-test), as well as comparisons between different differentiations (***P < 0.001, ****P < 0.0001, one-way ANOVA Tukey’s multiple comparison test; only significant comparisons are shown). **e**, Bar plots showing percentage of INS^+^ cells generated from VFGp3 or VFGp6 cultures derived from HUES4 (triangles) and H9 (circles) ESCs. Data are represented as mean ± s.e.m.; *N* = 4 independent experiments. Statistical analysis was performed for differentiation of each indicated cell type (**P < 0.01, ****P < 0.0001, unpaired two-tailed *t*-test), as well as comparisons between different differentiations (****P < 0.0001, unpaired two-tailed *t*-test; only significant comparisons are shown). **f**, Representative immunostaining (from three independent experiments) of VFGp6-derived β-like cells, stained with PDX1, INS and DAPI. Scale bar, 50 µm. **g**, Expression analysis of ESC-derived VFG cultures at different passages: RT–qPCR of the indicated genes in transient ADE and VFGs. Expression is normalized with *ACTB*. Data are represented as mean ± s.e.m.; *N* = 6 independent experiments. *P < 0.05, **P < 0.01, ***P < 0.001, ****P < 0.0001 (one-way ANOVA Dunnett’s multiple comparison test compared with ADE; only significant comparisons shown). *P < 0.05 (one-way ANOVA Dunnett’s multiple comparison test compared with VFGp3-4; only significant comparisons shown). Source data

**Extended Data Fig. 3:**
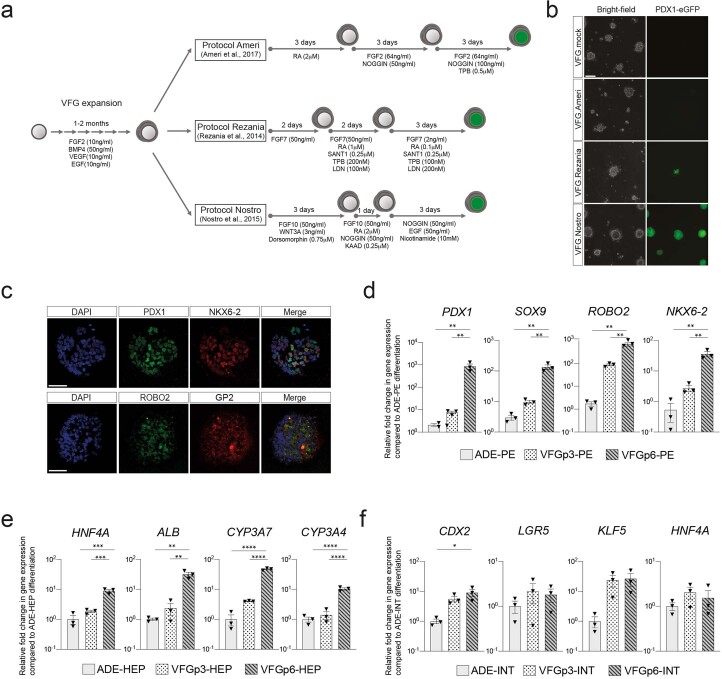
*in vitro* differentiation of VFG culture towards pancreatic, hepatic, and intestinal endoderm. **a**, Schematic diagram for stepwise pancreatic differentiation with protocols from Ameri et al.^22^, Rezania et al.^12^ and Nostro et al.^10^ from established VFG culture. **b**, Representative bright-field (left) and fluorescent (right) images for the PDX1-eGFP reporter VFGs differentiated with protocols indicated. Images represent three independent experiments. Scale bar = 50 μm. **c**, Representative immunostaining of PDX1 (green) and NKX6-2 (red) in the top row; ROBO2 (green) GP2 (red) in the bottom row, including DAPI (blue) for p6 VFG cells differentiated with protocol from Nostro et al.^10^. Images represent three independent experiments. Scale bar = 50 μm. **d**, Bar plot showing relative fold change in mRNA of pancreatic markers *PDX1*, *SOX9*, *ROBO2*, and *NKX6-2* in pancreatic differentiation from ADE and VFG at p3 and p6 cells. Data are represented as mean ± SEM (*N* = 3 independent experiments). **P < 0.01, (one-way ANOVA Tukey’s multiple comparison test, only significant comparisons are shown). **e**, Bar plot showing relative fold change in mRNA of hepatic markers *HNF4A*, *ALB*, *CYP3A7*, and *CYP3A4* in hepatic differentiation from ADE and VFG at p3 and p6 cells. Data are represented as mean ± SEM (*N* = 3 independent experiments). **P < 0.01, ***P < 0.001, ****P < 0.0001 (one-way ANOVA Tukey’s multiple comparison test, only significant comparisons are shown). **f**, Bar plot showing relative fold change in mRNA of intestinal markers *CDX2*, *LGR5*, *KLF5*, and *HNF4A* in hepatic differentiation from ADE and VFG at p3 and p6 cells. Data are represented as mean ± SEM (*N* = 3 independent experiments). *P < 0.05 (one-way ANOVA Tukey’s multiple comparison test, only significant comparisons are shown). Comparisons without an indicated *P* value are not significant. Source data

To compare the differentiation efficiency of expanding VFGs with standard differentiation, we employed aspects of three established protocols for the derivation of pancreatic endoderm (PE) from ESCs^10,12,22^ (Extended Data Fig. 3a). In two of these protocols^12,22^ we observed relatively inefficient differentiation (<20% PDX1^+^) (Fig. 2a and Extended Data Fig. 3b). However, a protocol coupling BMP inhibition, FGF and WNT activation^10^ resulted in >80% PDX1^+^ induction, suggesting that VFG cultures are adapted to protocols harnessing signals regulating ventral pancreatic specification. VFG-derived PE expressed pancreatic markers including PDX1 and NKX6-2, Glycoprotein 2 (GP2) (refs. ^22,25^) and the ventral pancreatic marker Roundabout2 (ROBO2) (ref. ^26^) (Extended Data Fig. 3c). Consistent with the observation that ventral pancreatic bud expands more than the dorsal bud^18^, cells differentiated via this third protocol, and not the other two, proliferate (Fig. 2b,c).

The efficiency of pancreatic differentiation increased with time in expansion and was maintained at a similar level following six passages (Fig. 2d and Extended Data Fig. 3d). Later passage VFG cells re-introduced into differentiation also produced more insulin-positive (INS^+^) endocrine cells (Fig. 2e,f). Similarly, extended VFG expansion produced enhanced hepatic, but not intestinal, differentiation (Extended Data Fig. 3e,f). Expression of primitive-streak and early endoderm genes, *GSC*, *GATA6* and *CER1*, decreased upon expansion (Fig. 2g). General endoderm markers expressed in the ventral foregut, such as *FOXA2*, *HHEX* and *SOX17*, were expressed throughout expansion at levels comparable to those in transient ADE cells. Expression of the foregut marker *HNF1B*^27^ and the ventral foregut markers *TBX3*, *ID2* and *GATA3* (refs. ^15,28,29^) were elevated in early passaged (p3 and p4) VFG cells and maintained during expansion. The pancreatic progenitor marker *PDX1* was never detected during VFG expansion.

### Chromatin accessibility is fine-tuned in VFG expansion

**Fig. 3:**
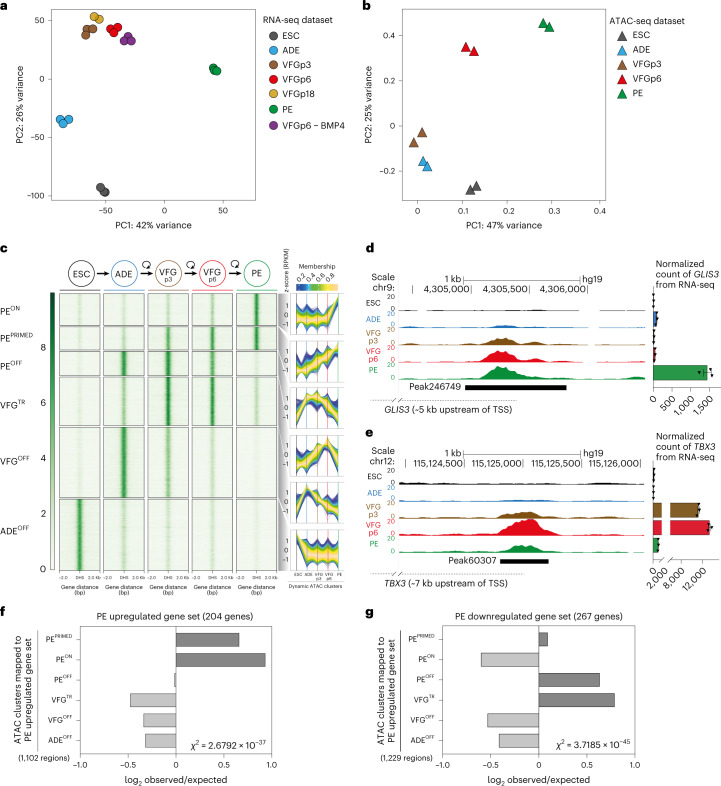
Dynamic chromatin accessibility and gene expression during VFG expansion and pancreatic differentiation. **a**, PCA based on top 2,000 differentially expressed genes in bulk RNA-seq dataset (from three or two (VFGp18) independent experiments) of ESC, transient ADE and VFG cells (at p3, p6 and p18), VFG cells cultured without BMP4 (at p6) and PE cells generated from VFGp6 cells. **b**, PCA of ATAC-seq dataset (from two independent experiments) for ESC, transient ADE and VFG cells (at p3 and p6). **c**, Left: heat maps of the normalized ATAC-seq signal for the dynamic clusters identified by fuzzy clustering. DHS is defined as a peak of Tn5 insertions in ATAC-seq. Right: Time-course sequencing (TC-seq) trajectories for each cluster. Membership score reflects how well a given enhancer follows the pattern identified in time-course analysis. **d**,**e**, Left: representative UCSC Genome Browser screenshot (from two independent experiments) at the *GLIS3* (**d**) and *TBX3* (**e**) locus showing ATAC-seq data from ESC, ADE, VFGp3, VFGp6 and PE. Genome coordinates (bp) are from the hg19 assembly of the human genome. The PE^PRIMED^ regulatory element (peak246749) (**d**) and VFG^TR^ element (peak60307) (**e**) are shown with a black bar. Approximate distance between the element and the respective TSS is indicated by a broken dashed line in each panel. Right: RNA-seq data (normalized read count) for *GLIS3* (**d**) and *TBX3* (**e**) across the same conditions as the ATAC tracks. RNA-seq data are represented as mean ± s.e.m.; *N* = 3 independent experiments. **f**,**g**, Bar plot showing enrichment scores (log_2_ observed/expected) of ATAC peak sets found within a 200 kb window from genes upregulated (**f**) or downregulated (**g**) between PE and VFGp6 across the defined ATAC peak clusters. Genes considered here had a base mean expression >1,000, log_2_FC > 1.5 and adjusted *P* < 0.05. For annotation, see Supplementary Table 1d–g. Analysis using lower base mean (100) or reduced genomic window sizes (25 kb) are shown in Extended Data Fig. 3g–j. All data shown are significant using chi-squared analysis. Source data

**Extended Data Fig. 4:**
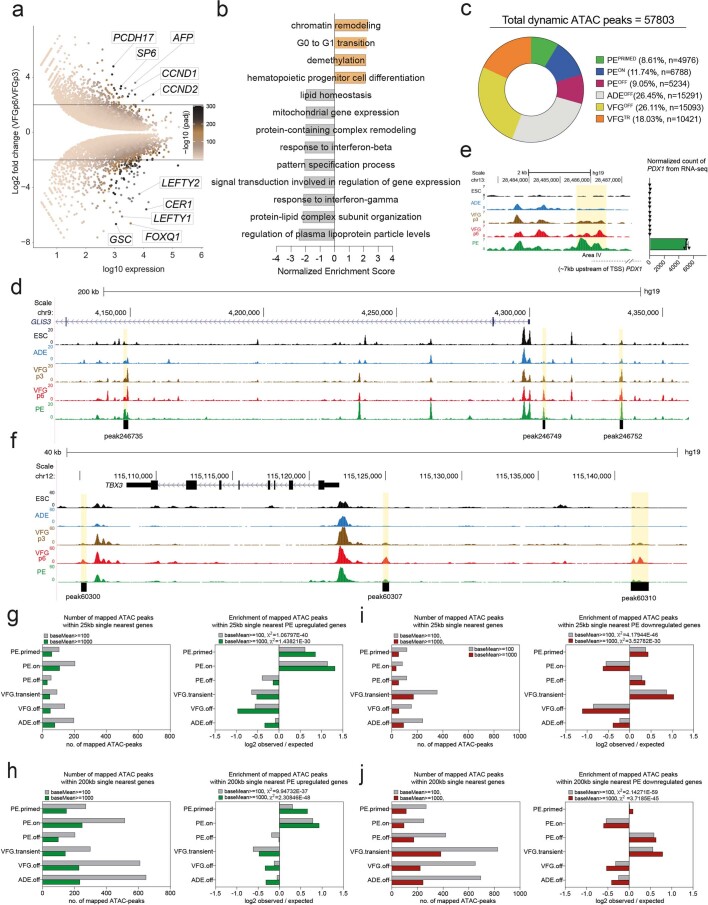
The dynamic chromatin landscape and gene expression in VFG expansion and further differentiation. **a**, MA-plot representing differential expression in VFGp6 versus VFGp3 culture (Log2 fold change > 2, *P* < 0.05) (*N* = 3 independent experiments). **b**, GSEA for GO-BP of VFGp6 compared to VFGp3 cells. Normalized Enrichment Score for significant terms for VFGp6 are shown as positive value, and VFGp3 as negative value (FDR < 0.05). **c**, Pie-chart showing distribution of dynamic ATAC-peaks (n = 57803) with percentage and numbers of peak indicated per cluster in Fig. 3c. **d**, Representative UCSC Genome Browser screen shot (from two independent experiments) at the *GLIS3* locus showing ATAC-seq data from ESC, ADE, VFGp3, VFGp6, and PE. Genome coordinates (bp) are from the hg19 assembly of the human genome. PE^PRIMED^ elements (peaks 246735, 246749, and 246752) are shown at the bottom and the corresponding regions are highlighted in yellow. **e**, Left: Representative UCSC Genome Browser screen shot as in d. The region of the area IV enhancer is highlighted in yellow. Approximate distance between the region and *PDX1* TSS is indicated by a broken dashed line. Right: bar plots for expression (normalized RNA-seq counts, *N* = 3 independent experiments) for *PDX1* RNA across the same samples as ATAC-seq. **f**, Representative UCSC Genome Browser screen shot at the *TBX3* locus as in d. VFG^TR^ elements (peaks 60300, 60307, and 60310) are shown at the bottom and the corresponding regions are highlighted in yellow. **g-h**, Mapping dynamic enhancer classes to gene expression (up-regulated genes). Left: Number of mapped ATAC peaks in each cluster defined in Fig. 3c located within 25 Kb (**g**) or 200 Kb (**h**) of the single nearest gene’s TSS from the PE up-regulated gene set with baseMean >100 (grey) or >1000 (green). Right: Enrichment (log2 observed/expected) of the PE up-regulated gene set with baseMean > 100 (grey) or >1000 (green), in proximity (within a 25 or 200 Kb window) to ATAC-clusters defined in Fig. 3c. **i-j**, Mapping dynamic enhancer classes to gene expression (down-regulated genes) for elements located within 25 Kb (**i**) or 200 Kb (**j**) of the single nearest gene’s TSS from the PE down-regulated gene set, analysis and labels as in **g**. All data shown are significant by chi-square analysis. Source data

Principal component analysis (PCA) of VFG RNA-seq data at multiple passages showed that VFG cells form a cluster separated from ADE, PE and ESC (Fig. 3a). Different passages of VFGs, cultured with and without BMP4, cluster together and separate in the first principal component from PE. Comparison between VFG passage (p)3 and VFGp6 cells shows a small set of genes (21 upregulated and 102 downregulated) with significant changes in expression (log_2_ fold change (FC) > 2, *P* < 0.05), including downregulated primitive-streak markers (*GSC*, *CER1* and *LEFTY1*) (Extended Data Fig. 4a and Supplementary Table 1a). The only Gene Ontology terms for gene set enrichment with expansion were associated with chromatin modification and cell-cycle transition (Extended Data Fig. 4b). We used assay for transposase-accessible chromatin using sequencing (ATAC-seq) to map chromatin accessibility during the progression of hESCs to pancreatic progenitors, at five defined stages of differentiation and expansion: hESC, ADE, VFGp3, VFGp6 and PE. Unlike the transcriptome of different passage VFG cultures that cluster together by PCA, we observed considerable change in the ATAC-seq profile as a function of time in culture, with the higher-passage VFGs moving towards PE (Fig. 3b).

We used general linear modelling^30^ to define the dynamic changes in chromatin accessibility at promoter-distal ATAC-seq peaks (putative enhancers) across these five stages of differentiation. This resulted in a dynamic set of 57,803 sites (Extended Data Fig. 4c) showing chromatin opening or closing in at least one stage of differentiation. Temporal patterns of chromatin accessibility were defined using *c*-means clustering, producing eight clusters corresponding to six distinct groups of putative enhancers (Fig. 3c and Supplementary Table 1b). The largest group of sites are where chromatin accessibility is reduced at the start of differentiation and remains closed for the duration through to PE. The VFG^OFF^ cluster contains sites that become accessible during ESC to ADE differentiation, but that then lose accessibility during VFG differentiation or expansion, so that they are inaccessible in PE. The PE^OFF^ cluster also appears at ADE and then loses accessibility but only after VFG expansion. The PE^ON^ cluster encompasses regions that only open up during differentiation to PE. We defined two VFG clusters, VFG transient (VFG^TR^) and PE^PRIMED^ clusters.

Chromatin accessibility for the PE^PRIMED^ cluster increases gradually during VFG expansion and is most accessible in PE. An element located ~5 kb upstream of the *GLIS3* transcriptional start site (TSS) (Fig. 3d, left, and Extended Data Fig. 4d) is an example of this. In vivo *Glis3* is expressed in pancreatic endocrine progenitors and then beta cells^31^. RNA-seq shows that *GLIS3* is not expressed until PE differentiation from expanded VFGs (Fig. 3d, right). We also observed increases in accessibility in the conserved enhancer regions (area IV) of *PDX1* (refs. ^32,33^) (Extended Data Fig. 4e). The VFG^TR^ cluster contains regions where chromatin accessibility increases during VFG expansion and is then shut down during differentiation to PE. The putative enhancers located ~7 kb upstream of the *TBX3* TSS (Fig. 3e, left, and Extended Data Fig. 4f) are an example of this. *TBX3* is expressed in the developing human posterior foregut (FG) and liver bud progenitors^20,34^ and is expressed specifically in VFGs, but then silenced during the differentiation to PE (Fig. 3e, right).

To link these enhancer clusters to changes in gene expression, we defined significantly changing genes in the transition from expansion into further differentiation (log2FC > 1.5, *P* < 0.05) (Supplementary Table 1c). To pair enhancers with specific genes, we considered enhancers located either within 25 kb or 200 kb of the single nearest gene’s TSS and we excluded low level changes in basal gene expression (Supplementary Table 1d,e). While filtering out gene expression noise that occurs with passaging reduces the size of the gene set, we were able to define enhancers located within either 25 kb or 200 kb of upregulated PE genes. Regardless of which enhancer set used, we observed significant enrichment of both PE^PRIMED^ and PE^ON^ enhancer classes with upregulated PE genes (Fig. 3f and Extended Data Fig. 4g,h), although the enrichment is greater for enhancers located closest to the genes they regulate. We also identified enhancers at the same distance from genes downregulated in differentiation (Supplementary Table 1f,g). These downregulated PE gene sets were associated with the PE^OFF^ and VFG^TR^ enhancer categories (Fig. 3g and Extended Data Fig. 4i,j). Taken together, this suggests that VFG expansion primes some pancreatic enhancers for later target gene induction while decommissioning enhancers driving gene expression inappropriate for the PE lineage.

### Differentiation imperfectly realizes the VFG enhancer landscape

**Extended Data Fig. 5:**
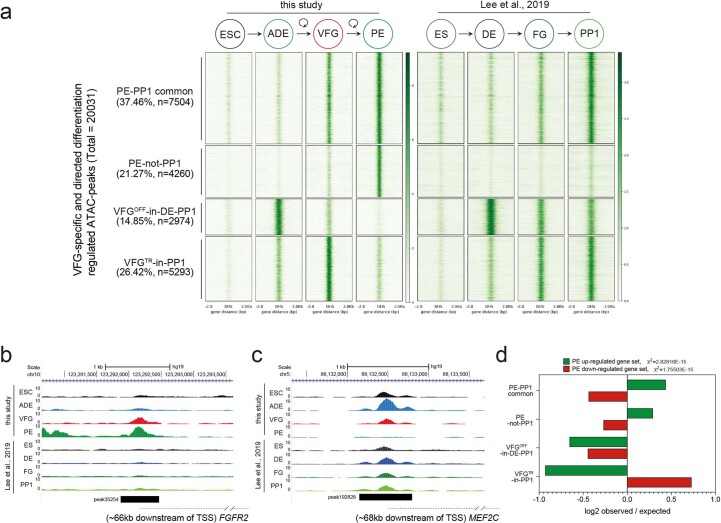
VFG expansion enables consolidation of an enhancer landscape that is imperfectly realized during directed differentiation. **a**, A comparison of chromatin accessibility of enhancers charted in this study (heatmap, left) with the Lee et al. dataset^35^ (heatmap, right). Enhancers in the group “PE-PP1 common” are the pancreatic endoderm enhancers that are activated independent of VFG expansion (37.46%, n = 7504). Enhancers in the group PE-not-PP1 are PE enhancers that are activated only if PE is differentiated from expanding VFGs (21.27%, n = 4260). Enhancers in the “VFG^OFF^-in-DE-PP1” group, represent a subset of ADE enhancers that are inactivated during VFG expansion (14.85%, n = 2974). The “VFG^TR^-in-PP1” enhancer group at the bottom of the heatmap (26.42%, n = 5293) are inactivated in PE derived from expanding VFGs, but not in directed differentiation. **b-c**, Representative UCSC Genome Browser screen shot (from two independent experiments) showing examples of a PE-not-PP1 enhancer (peak35254), in an intron of the *FGFR2* locus (**b**) and a VFG^OFF^-in-DE-PP1 group (peak192828) contained within an intron of the *MEF2C* locus (**c**). Approximate distance between elements and TSS is indicated by a broken dashed line in each panel. **d**, Bar plot showing the prevalence (log2 observed/expected) of ATAC peaks within a 200 Kb window from genes up-regulated (green) or down-regulated (red) between PE and VFGp6 across the defined ATAC peak clusters. Genes considered have a base mean expression > 1000, log2 fold change > 1.5 and *P* < 0.05. All data shown are significant in chi-square analysis. Source data

To understand the extent to which the enhancer network induced during expansion is normally exploited in directed differentiation, we compared our data with a previous study that profiled chromatin accessibility by ATAC-seq during the differentiation of hESC through DE and posterior FG stages to pancreatic progenitors (PP1) (ref. ^35^). On the basis of this analysis we could define a common set of putative enhancers activated in either VFGs or FG^35^ and that then remain accessible in later differentiation (PE or PP1), respectively (PE-PP1 common); and a class of element that is not induced in the absence of expansion (PE-not-PP1) (Extended Data Fig. 5a,b and Supplementary Table 2a). Many of the peaks that closed down during or after VFG expansion (VFG^OFF^-in-DE-PP1 and VFG^TR^-in-PP1) remain accessible in the FG or PP1 stages (Extended Data Fig. 5a,c). Together, these data suggest that VFG expansion allows for the commissioning of enhancers relevant to pancreatic differentiation and the decommissioning of enhancers for alternative lineages. This process appears bypassed in directed differentiation.

Mapping of these enhancer elements to potential target loci (located within 200 kb) (Supplementary Table 2b,c) reveals an enrichment for the two pancreatic endoderm enhancer clusters, PE-PP1 common and PE-not-PP1, in the vicinity of genes upregulated in VFG-derived PE (the same gene set used for Fig. 3f,g) (Extended Data Fig. 5d). However, elements induced in directed differentiation, but not active in VFG-derived PE (VFG^OFF^-in-DE-PP1 or VFG^TR^-in-PP1), do not correlate with our PE upregulated gene set. Moreover, the PE downregulated gene set correlates with VFG^TR^-in-PP1 elements. These observations suggest that expansion is required for appropriate enhancer decommissioning.

**Extended Data Fig. 6:**
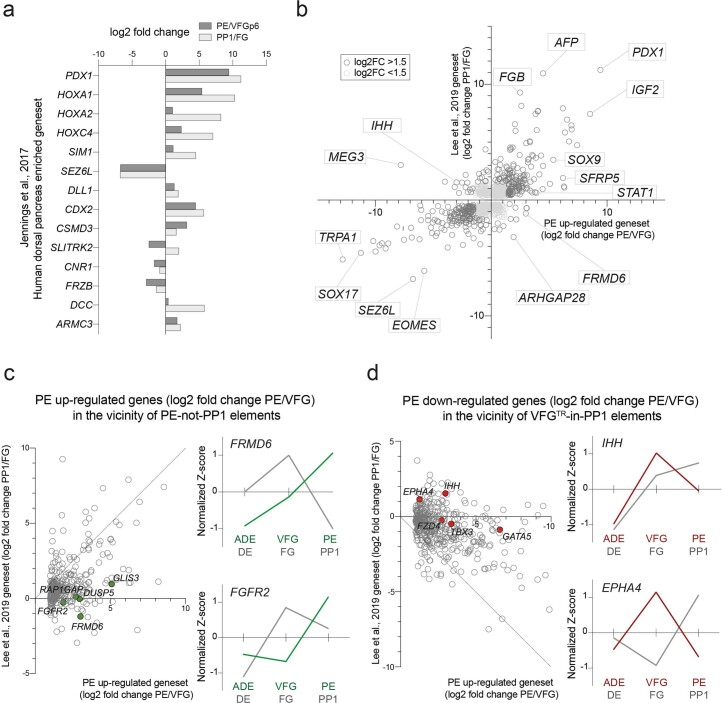
Characterization of global transcriptional changes between PE/VFG and PP1/FG cells. **a**, Bar plot showing differential expression of 13 dorsal pancreas markers (log2 fold change) in PE/VFGp6 and PP1/FG from RNA-seq dataset (*N* = 3 independent experiments). DHS is defined as a peak of Tn5 insertions in ATAC-seq. **b**, Scatter plot of differential expression for gene regulated in PE/VFGp6 (horizontal axis) and in PP1/FG (vertical axis) (*N* = 3 independent experiments). **c-d**, Left: Scatter plot of differential expression for genes up-regulated (**c**) or down-regulated (**d**) in PE vs VFG (as defined in Extended Data Fig. 5d), and within 200Kb of minimum one ATAC peak in the PE-not-PP1 (**c**) or VFG^TR^-in-PP1 (**d**) clusters respectively, vs their expression after directed differentiation (PP1/FG). The diagonal line indicates where there is no difference in differential expression between two comparisons (datasets). Right: normalized z-score expression of representative candidates (**c**: *FRMD6* and *FGFR2* and **d**: *IHH* and *EPHA4*). Normalized z-score expression for each candidate was plotted for the ADE, VFG (p6), and PE conditions (green in **c** and red in **d**), and the DE, FG, and PP1 conditions (grey) (*N* = 3 independent experiments).

In embryogenesis, the pancreas is derived from two buds that originate in different regions of the posterior FG, dorsal and ventral^6^. As the ventral pancreas is derived from the ventral foregut, we assessed the expression of markers thought to distinguish the dorsal pancreatic lineages^36^. Extended Data Fig. 6a shows the increase in expression of these markers in directed differentiation as foregut-like cells give rise to PP1 and suggests that directed differentiation has more of a dorsal identity.

To explore global correlations between genes differentially regulated in pancreatic endoderm derived from VFGs and directed differentiation, we plotted gene expression from both protocols (Extended Data Fig. 6b) and focused on the two classes of expansion dependent elements, PE-not-PP1 and VFG^TR^-in-PP1 (Extended Data Fig. 6c,d, left). Genes in the vicinity of PE-not-PP1 elements are better induced in VFG-derived PE than directed differentiation, whereas genes mapped to elements decommissioned as a result of expansion—VFG^TR^-in-PP1—are more extensively downregulated when PE is differentiated from expanding VFGs. Examples of expansion-dependent upregulation include *FRMD6* and *FGFR2* and for those ectopically expressed in directed differentiation, *IHH* and *EPHA4* (Extended Data Fig. 6c,d, right). These analyses suggest that there are differences in messenger RNA expression related to expansion dependent changes in enhancer accessibility.

### VFG expansion captures human foetal organ-specific enhancers

**Extended Data Fig. 7:**
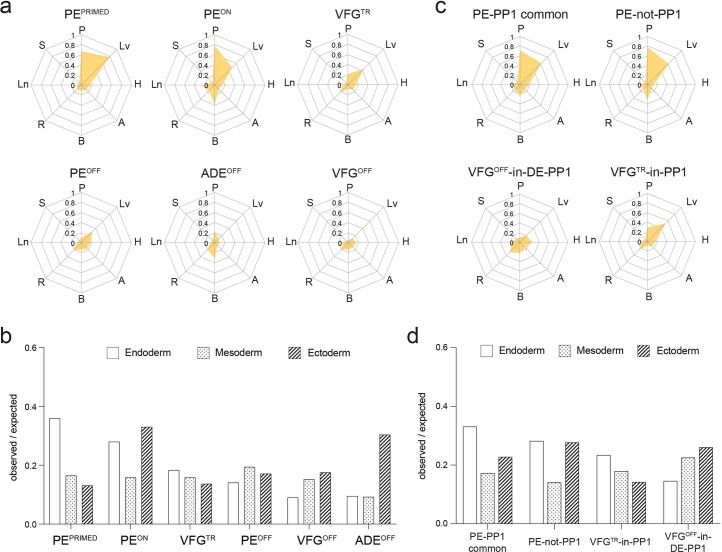
VFG expansion insures higher fidelity regulation of enhancers normally exploited in fetal organogenesis. **a-b**, Enrichment of tissue-specific (**a**) and lineage-specific (**b**) H3K27ac enhancers from human embryos (from two independent experiments for most tissue types, except for stomach where only one sample was available) in different ATAC clusters defined in Fig. 3c were displayed by enrichment score (observed/expected) in radar charts (**a**) and in bar plot (**b**). **c-d**, Enrichment of tissue-specific (**c**) and lineage-specific (**d**) H3K27ac enhancers from human embryos (from two independent experiments for most tissue types, except for stomach where only one sample was available) across different VFG-specific ATAC clusters defined in Extended Data Fig. 4a by enrichment score (observed/expected) in a radar chart (**c**) and bar plot (**d**). P: pancreas, Lv: liver, H: heart, A: adrenal, B: brain, R: RPE, Ln: lung, S: stomach. Source data

To determine how the enhancer landscape captured during VFG expansion and PE differentiation in vitro corresponds with pancreatic development in vivo, we compared our ATAC-seq data with H3K27ac data obtained from micro-dissected endodermal (pancreatic, liver, lung and stomach), mesodermal (adrenal and heart) and ectodermal (retinal pigment epithelium (RPE) and brain) tissues collected from Carnegie stages 15–22 human embryos^37^ (Supplementary Table 3). Consistent with the VFG identity of our cultures, the PE^PRIMED^ class of element is enriched for both liver and pancreatic enhancers, while the PE^ON^ class overlaps more extensively with pancreatic elements (Extended Data Fig. 7a and Supplementary Table 4a). Enhancer clusters that shut down as expanded VFGs differentiate to PE (VFG^TR^ and PE^OFF^) are most enriched for enhancers active in the developing liver, consistent with their role in non-pancreatic VFG differentiation. Elements decommissioned in early differentiation or expansion (ADE^OFF^ or VFG^OFF^) are non-VFG enhancers, including elements spanning the ectodermal and mesodermal lineages (Extended Data Fig. 7b and Supplementary Table 4a,b).

We assessed how the enhancers classes that differ between in vitro VFG expansion and direct differentiation from pluripotent cells compare with human organogenesis. Not surprisingly, the PE-PP1 common class of element was enriched in enhancers accessible in the ventral-foregut-derived pancreas and liver, while expansion-dependent PE-not-PP1 enhancers were more enriched in pancreatic elements (Extended Data Fig. 7c,d). Moreover, the set of enhancers accessible in directed differentiation, but decommissioned as consequence of expansion (VFG^OFF^-in-DE-PP1) or VFG differentiation to PE (VFG^TR^-in-PP1), did not contain meaningful numbers of pancreatic elements.

### Enhancers explicitly correlating with VFG proliferation

**Fig. 4:**
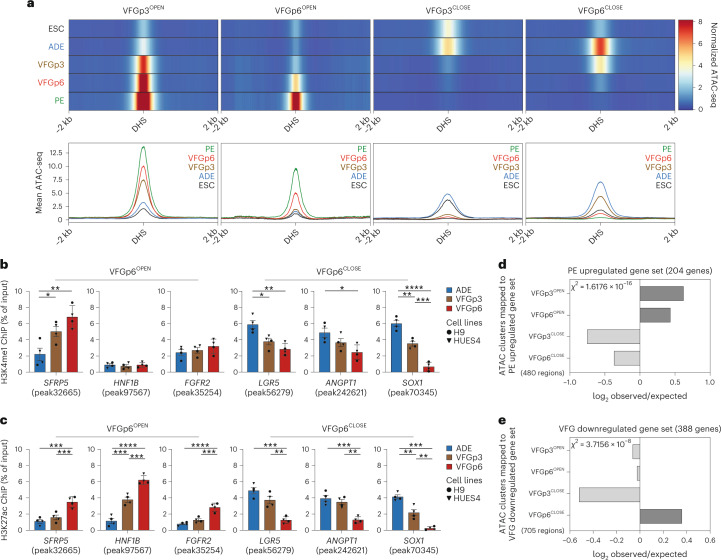
VFG proliferation-dependent enhancers are associated active histone marks and correlate with later gene expression. **a**, Enhancer classification relative to VFG expansion time (from two independent experiments). Top: heat maps of normalized ATAC-seq signal in enhancers that open (VFGp3^OPEN^ and VFGp6^OPEN^) or close (VFGp3^CLOSE^ and VFGp6^CLOSE^) at VFGp3 or p6. VFGp3^CLOSE^ group comprises ADE enhancers that are shut down during VFG expansion at passage 3. Bottom: average ATAC-seq signal in 10 bp bins for these enhancers in same stages. **b**,**c**, H3K4me1 (**b**) and H3K27ac (**c**) enrichment by ChIP–qPCR for ADE, VFGp3 and p6 culture at VFGp6^OPEN^ enhancer regions: *SFRP5* (peak32665), *HNF1B* (peak97567) and *FGFR2* (peak35254); and at VFGp6^CLOSE^ enhancer regions of *LGR5* (peak56279), *ANGPT1* (peak242621) and *SOX1* (peak70345). Circles and triangles mark cells derived from H9 and HUES4 WT ESCs, respectively. Data are represented as mean ± s.e.m.; *N* = 4 independent experiments. *P < 0.05, **P < 0.01, ***P < 0.001, ****P < 0.0001 (one-way ANOVA Tukey’s multiple comparison test; only significant comparisons shown). **d**,**e**, Bar plot showing the prevalence (log_2_ observed/expected) of ATAC peaks within a 200 kb window from genes upregulated between PE and VFGp6 (**d**) and from genes downregulated between ADE and VFGp6 (**e**) across the ATAC peak clusters (defined in **a**). Genes considered here had a base mean expression >1,000, absolute log_2_FC > 1.5 and adjusted *P* < 0.05. All data shown are significant using chi-squared analysis. Source data

Although differentiation efficiency increased with time in VFG culture, we wished to exclude alterations to enhancer accessibility that could result from the shift to VFG culture and variations in pancreatic differentiation arising between the dorsal and ventral lineages. We therefore defined a restricted set of enhancers specifically regulated between passages 3 and 6, correlating with enhanced pancreatic and hepatic, but not intestinal, differentiation. We segregated defined enhancers activated or inactivated for the first time at passage 3 (VFGp3^OPEN^ and VFGp3^CLOSE^) and those responding to increased passaging (VFGp6^OPEN^ and VFGp6^CLOSE^) (Fig. 4a). While the chromatin accessibility of VFGp3^OPEN^ and VFGP3^CLOSE^ enhancer elements also respond to expansion, the influence of passaging is difficult to resolve from an initial response to the change in culture medium.

To investigate whether there was a change in chromatin state of enhancers specifically responding to expansion, we performed chromatin immunoprecipitation (ChIP)–quantitative polymerase chain reaction (qPCR) for H3K27 acetylation (H3K27ac) and H3K4 monomethylation (H3K4me1) for multiple expansion-regulated elements (Fig. 4b,c). There were robust changes in H3K27ac deposition at these elements between VFGp3 and VFGp6, while changes in H3K4me1 were more subtle. We also paired these explicitly expansion-dependent enhancers to specific genes (within 200 kb of the single nearest gene’s TSS) (Supplementary Table 5a). We identified 480 enhancers explicitly correlated with expansion and located them within 200 kb of PE upregulated genes (the same gene set being used in Fig. 3f) (Supplementary Table 5b). Chromatin accessibility at both VFGp3^OPEN^ and VFGp6^OPEN^ enhancers correlated with gene expression (Fig. 4d). Similarly, for genes downregulated during VFG expansion (log_2_FC < −1.5, *P* < 0.05), we observed good correlation with decommissioning (Supplementary Table 5c–e), where in this instance, only the expansion-specific VFGp6^OFF^ correlates well with gene expression (Fig. 4e) here.

**Fig. 5:**
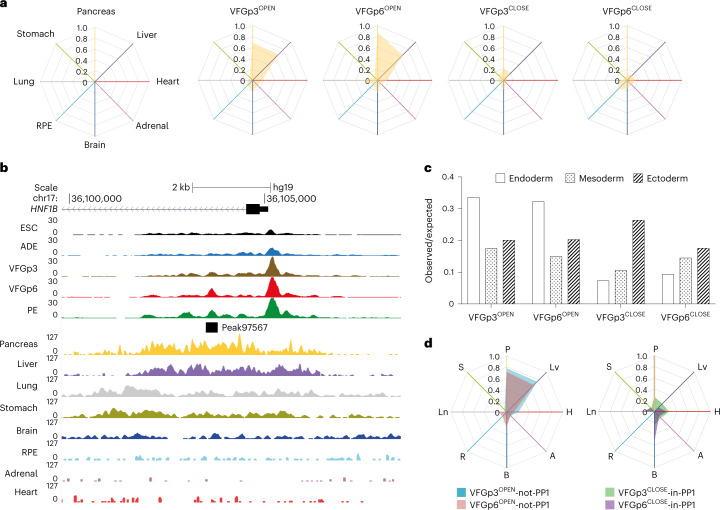
VFG expansion captures enhancers that are active during human ventral foregut-derived organogenesis. **a**, Enrichment of tissue-specific H3K27ac enhancers from human embryos (from two independent experiments for most tissue types, except for stomach where only one sample was available) in different ATAC clusters defined in Fig. 4a displayed by enrichment score (observed/expected) in radar charts. **b**, Representative UCSC Genome Browser screenshot (from two independent experiments) at the *HNF1B* locus showing ATAC-seq data from this study (ESC, ADE, VFGp3, VFGp6 and PE) and H3K27ac ChIP–seq data^37^ from multiple human embryonic tissues (pancreas, liver, lung, stomach, brain, RPE, adrenal and heart). Genome coordinates (bp) are from the hg19 assembly of the human genome. VFGp6^OPEN^ (peak97567) element overlapping with pancreatic-specific H3K27ac enhancer is shown at the bottom, and the approximate distance between the elements and the *HNF1B* TSS is indicated. **c**, Enrichment of lineage-specific H3K27ac enhancers (endoderm, ectoderm and mesoderm) from human embryos^37^ in the different VFG expansion-specific ATAC clusters defined in Fig. 4a by enrichment score (observed/expected). **d**, Enrichment of tissue-specific H3K27ac enhancers from human embryos across different VFG^OPEN^ and VFG^CLOSE^ clusters (defined in Fig. 4a) that are not regulated in directed differentiation were displayed by enrichment score (observed/expected) in radar charts. P, pancreas; Lv, liver; H, heart; A, adrenal; B, brain; R, RPE; Ln, lung; S, stomach. Source data

To ask whether enhancers correlating directly with expansion are also related to ventral foregut specific differentiation, we compared these enhancers with the in vivo regulatory landscape in foetal organ development (Supplementary Table 6). Consistent with the interpretation that extended VFG culture lays the groundwork for further differentiation, both the VFGp3^OPEN^ and the expansion-specific VFGp6^OPEN^ clusters overlap with active enhancer sets from the foetal pancreas and liver, but not stomach, lung or other non-endodermal organs (Fig. 5a,b). Both sets of VFG^OPEN^ enhancers are enriched in the endoderm lineage, while the VFG^CLOSED^ enhancers contain more mesodermal and ectodermal elements (Fig. 5c). Finally, we compared expansion clusters with directed-differentiation clusters (Fig. 5d). Both VFG^OPEN^ enhancer clusters that are not regulated in directed differentiation (VFGp3^OPEN^ and VFGp6^OPEN^-not-PP1) overlap with foetal pancreas and liver enhancers sets, while VFG decommissioned enhancers that remain accessible in directed differentiation (VFGp3^CLOSED^ and VFGp6^CLOSED^-in-PP1) have little in common with pancreatic and hepatic elements.

### TFs FOXA and HHEX in pancreatic priming

**Fig. 6:**
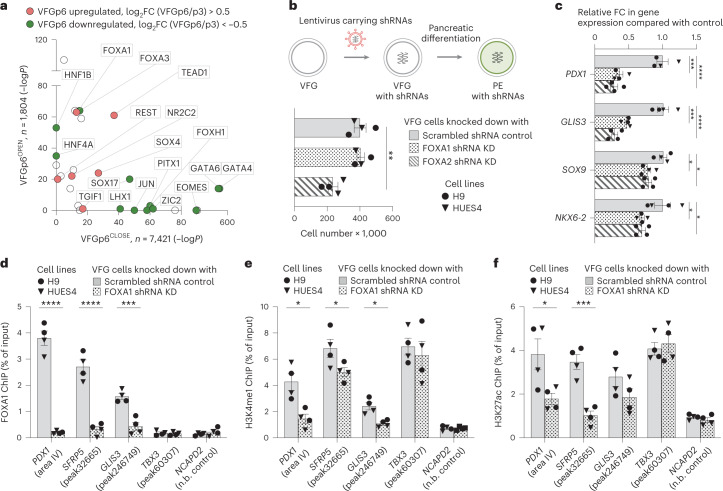
FOXA proteins are required for VFG enhancer priming towards pancreatic differentiation. **a**, TF motif enrichment in VFGp6^OPEN^ (*n* = 1,804) and VFGp6^CLOSE^ (*n* = 7,421) ATAC clusters; *n*, number of peaks analysed. *P* values were derived from hypergeometric enrichment using HOMER default background. Candidate factors with *P* > 1 × 10^−10^ for both clusters were not included in the plot. Gene expression of the candidate factors that upregulated (red) or downregulated (green) from VFGp3 to VFGp6 (log_2_FC > 0.5, *P* < 0.05) are labelled. **b**, Top: schematic of FOXA1 and FOXA2 shRNA KD VFG cells and their PE differentiation. Bottom: histogram for proliferation assay (cell counts) for FOXA1 and FOXA2 shRNA KD and scrambled shRNA control VFG cells. Data are represented as mean ± s.e.m.; *N* = 4 independent experiments. Statistical analysis was performed between KDs and control VFG cells (**P < 0.01, unpaired two-tailed *t*-test; only significant comparisons are shown). **c**, Differentiation of FOXA1 and FOXA2 shRNA KD and scrambled control VFG cells to PE, with legend shown in **b**. Relative FC in mRNA of pancreatic genes (*PDX1*, *GLIS3*, *SOX9* and *NKX6-2*) was assayed by RT–qPCR. Expression is normalized to *ACTB*. Data are represented as mean ± s.e.m.; *N* = 4 independent experiments. *P < 0.05, ***P < 0.001, ****P < 0.0001 (one-way ANOVA Dunnett’s multiple comparison test compared with control). **d**–**f**, FOXA1 binding (**d**), H3K4me1 (**e**) and H3K27ac (**f**) enrichment by ChIP–qPCR at enhancer regions of *PDX1* (area IV), *GLIS3* (peak246749) and *TBX3* (peak60307) in FOXA1 shRNA KD VFG and scrambled control cell lines. An intragenic region of *NCAPD2* served as a non-bound (n.b.) control. Data are represented as mean ± s.e.m.; *N* = 4 independent experiments. Statistical analysis was performed between the KD and control VFG cells. *P < 0.05, ***P < 0.001, ****P < 0.0001, (unpaired one-tailed *t*-test; only significant comparisons are shown). Source data

**Extended Data Fig. 8:**
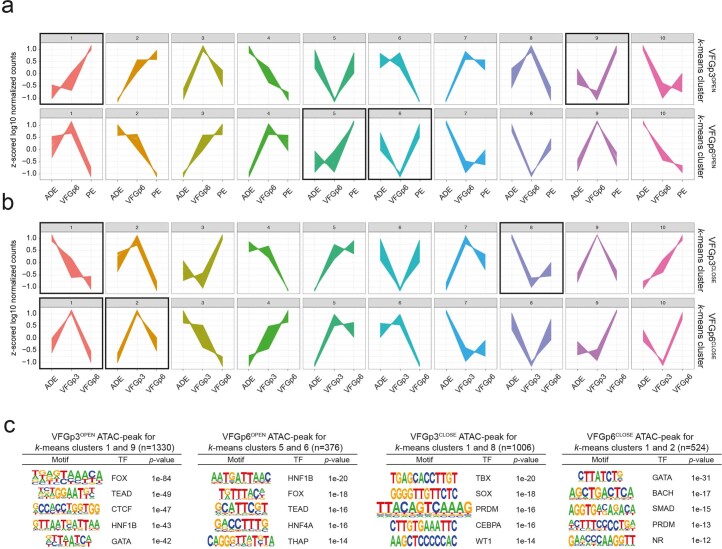
*k*-means clustering and motif analysis for VFG expansion dependent ATAC-clusters. **a-b**, *k*-means clustering of genes within 200Kb of peaks in ATAC clusters as defined in Fig. 4a (VFGp3^OPEN^, VFGp6^OPEN^, VFGp3^CLOSE^, and VFGp3^CLOSE^). Z-scored log10 normalized gene expression of ADE, VFGp6, and PE samples (**a**); and of ADE, VFGp3, and VFGp6 samples (**b**) were plotted for VFG^OPEN^ and VFG^CLOSE^ clustered genes respectively (n = 10). **c**, De novo motif search was made using Homer findMotifsGenome and searched within ±200 bp of peak center for genes mapped to the vicinity of VFGp3^OPEN^ (*k*-means clusters 1 and 9, n = 1330), VFGp6^OPEN^ (*k*-means clusters 5 and 6, n = 376), VFGp3^CLOSE^ (*k*-means clusters 1 and 8, n = 1006), and VFGp6^CLOSE^ (*k*-means clusters 1 and 2, n = 524).

To determine factors responsible for VFG enhancer priming, we assessed TF motifs in different enhancer classes (Fig. 6a and Supplementary Table 7), focusing on those linked directly to expansion, and regulated between P3 and P6. TF motifs in VFGp6^OPEN^ enhancers included FOXA factors and, to a lesser extent, a number of unrelated endodermal/hepatic factors broadly classed as hepatic nuclear factors (HNFs)^38^ and TEAD1. In contrast, motifs in VFGp6^CLOSED^ elements included early endoderm and mesendoderm factors such as GATA4,6 and EOMES. To further refine the association of specific TFs with these enhancer classes, we used *k*-means clustering to define patterns of mRNA expression associated with enhancers that are upregulated or downregulated during VFG expansion (Extended Data Fig. 8a,b) and selected clusters that correlated with differentiation. In those enhancers related to clusters of upregulated gene expression in pancreatic differentiation, we identified motifs for TF classes relevant to human pancreatic and liver development^36,39^, such as FOXA, HNF1B, TEAD and the architectural factor CTCF. For those enhancers mapping to downregulated clusters, we observed no motifs linked to pancreatic differentiation or function (Extended Data Fig. 8c).

**Extended Data Fig. 9:**
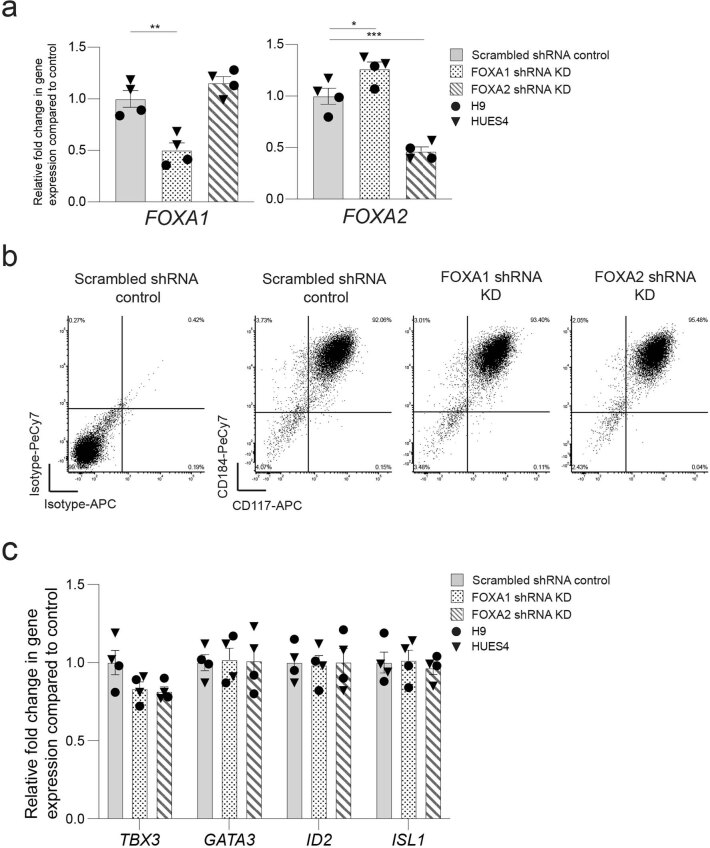
Characterization of FOXA1 and FOXA2 shRNA KD VFG cells. **a**, Expression analysis in FOXA1 and FOXA2 shRNA KD cells (described in Fig. 6b) by RT-qPCR. Expression of FOXA1 (left) and FOXA2 (right) in the KD cells was normalized relative to the expression in scrambled shRNA controls. Triangles and circles mark cells derived from HUES4 and H9 ESCs respectively. Data are represented as mean ± SEM (*N* = 4 independent experiments). *P < 0.05, **P < 0.01, ***P < 0.001 (unpaired two-tailed t-test). Comparisons without an indicated *P* value are not significant. **b**, Representative flow cytometry density plots showing CD184 (CXCR4) and CD117 (KIT) expression in scrambled shRNA control, FOXA1, and FOXA2 shRNA KD VFG cells. Bottom left quadrant indicates gating based on isotype staining controls in scrambled shRNA control VFG cells. Flow Cytometry plots represent three independent experiments. **c**, Expression analysis in FOXA1 and FOXA2 shRNA KD cells (described in Fig. 6b) by RT-qPCR. Expression of VFG markers *TBX3*, *GATA3*, *ID2*, and *ISL1* in the KD cells was normalized relative to that in scrambled shRNA controls. Triangles and circles mark cells derived from HUES4 and H9 ESCs respectively. Data are represented as mean ± SEM (*N* = 4 independent experiments). No statistical difference (unpaired two-tailed t-test) was found in the comparisons. Source data

Of TFs known to recognize FOX DNA binding motifs, embryonic expression patterns and phenotypes in mouse development suggest that FOXA1 and FOXA2 could be relevant to VFG-mediated enhancer priming^33,40^. FOXA2 is required for pancreas development and differentiation in both mouse^33^ and human ESCs^35^, and a requirement for FOXA1 in pancreas development is observed in the context of *FOXA1/2* double mutants. FOXA factors are known ‘pioneer TFs’ that access regulatory regions and prepare them for later activation^41^. However, *FOXA1* mutant ESCs undergo apparently normal directed pancreatic endoderm differentiation^35^. To assess their function in pancreatic priming during human VFG expansion, we knocked down FOXA1 and FOXA2 by shRNA during VFG expansion (Fig. 6b and Extended Data Fig. 9a). Neither factor produced a significant reduction in VFG marker expression (Extended Data Fig. 9b,c), although FOXA2, but not FOXA1, KD impaired VFG expansion. When VFG cells knocked down for either FOXA1 or FOXA2 were challenged in pancreatic differentiation, expression of pancreatic markers were significantly reduced (Fig. 6c). We confirmed FOXA1 binding, using ChIP–qPCR, at the *PDX1* enhancer area IV, the PE^PRIMED^ enhancers of *GLIS3*, and VFGp6^OPEN^ enhancer of *SFRP5*, but not in the VFG^TR^ element of *TBX3*. Binding was reduced in the stable FOXA1 KD VFG lines (Fig. 6d). KD of FOXA1 led to a significant reduction in H3K4me1 and H3K27ac at primed enhancers associated with *PDX1* and *SFRP5* (Fig. 6e,f), but not the enhancer associated with *TBX3*.

**Extended Data Fig. 10:**
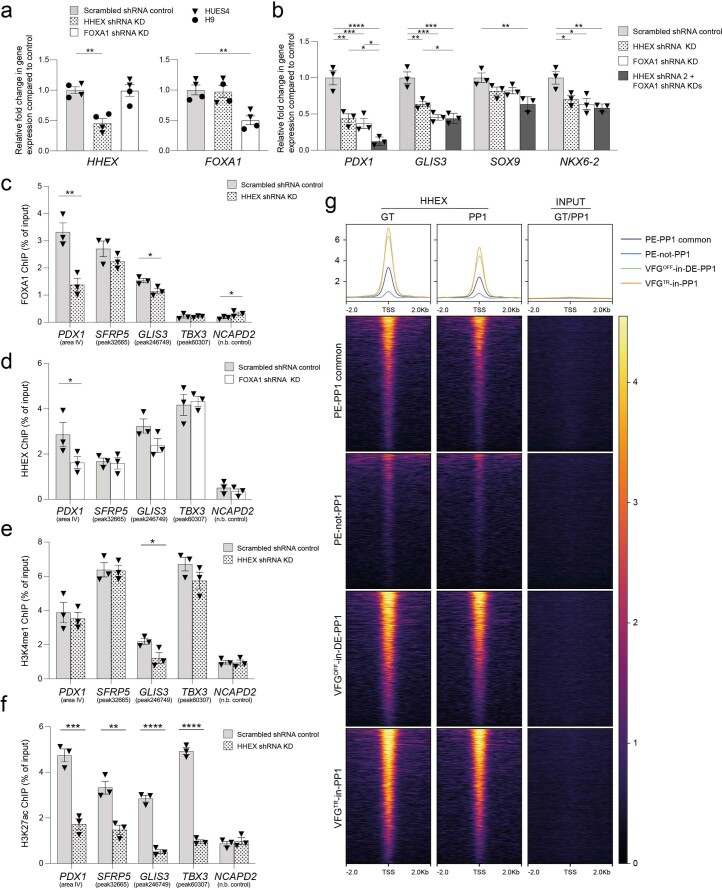
HHEX is also required alongside FOXA1 for enhancer priming in VFGs. **a**, RT-qPCR of HHEX (left) and FOXA1 (right) in the HHEX or FOXA1 KD cells. Expression was normalized relative to the scrambled shRNA controls. Triangles and circles mark cells derived from HUES4 and H9 ESCs respectively. Data are represented as mean ± SEM (*N* = 4 independent experiments). **P < 0.01 (unpaired two-tailed t-test, only significant comparisons are shown). **b**, Differentiation of scrambled control, HHEX KD, FOXA1 KD and HHEX/FOXA1 double KDs VFG cells to PE. Relative fold change of pancreatic genes (*PDX1*, *GLIS3*, *SOX9* and *NKX6-2*) was assayed by RT-qPCR and normalized relative to the scrambled shRNA controls. Data are represented as mean ± SEM (*N* = 3 independent experiments). *P < 0.05, **P < 0.01, ***P < 0.001, ****P < 0.0001 (one-way ANOVA Dunnett’s multiple comparison test compared with scramble control and HHEX/FOXA1 double KDs). **c-d**, FOXA1 (**c**) and HHEX (**d**) binding enrichment by ChIP-qPCR at enhancer regions of *PDX1* (area IV), *SFRP5* (peak32665), *GLIS3* (peak246749), and *TBX3* (peak60307) in HHEX and FOXA1 shRNA KD VFG and scrambled control cell lines. An intragenic region of *NCAPD2* served as non-bound control. Data are represented as mean ± SEM (*N* = 3 independent experiments). Statistical analysis was performed between KD and control VFG cells (*P < 0.05, **P < 0.01, unpaired one-tailed t-test, only significant comparisons are shown). **e-f**, H3K4me1 (**e**) and H3K27ac (**f**) enrichment by ChIP-qPCR at enhancer regions of *PDX1* (area IV), *SFRP5* (peak32665), *GLIS3* (peak246749), and *TBX3* (peak60307) in HHEX shRNA KD VFG and scrambled control cell lines. An intragenic region of *NCAPD2* served as a non-bound control. Data are represented as mean ± SEM (*N* = 3 independent experiments). Statistical analysis was performed between the KD and control VFG cells (*P < 0.05, **P < 0.01, ***P < 0.001, ****P < 0.0001, unpaired one-tailed t-test, only significant comparisons are shown). **g**, HHEX signal plotted on VFG-specific enhancer classes (PE-PP1 common, PE-not-PP, VFG^OFF^-in-DE-PP1, and VFG^TR^-in-PP1) at the FG and PP1 stages of directed differentiation (ChIP-seq dataset^42^) (from two independent experiments). Source data

HHEX is suggested to be an essential transcriptional regulator of directed ESC differentiation to pancreatic endoderm^42^. We therefore asked whether HHEX was required for expansion-linked pancreatic enhancer regulation. KD of FOXA1 or HHEX produced similar defects in pancreatic differentiation, and the double KD had a combinatorial effect on PDX1 induction (Extended Data Fig. 10a,b) consistent with the specific influence they have on each other’s binding at the PDX1 enhancer (Extended Data Fig. 10c,d). At VFG-linked enhancer elements, HHEX has a particularly pronounced effect on H3K27ac (Extended Data Fig. 10e,f). To gain insight into the relation of HHEX binding to our enhancer dataset, we aligned HHEX ChIP–seq data from directed differentiation^42^ to the enhancer regions from the different classes defined here (Extended Data Fig. 10g). HHEX binding at both direct differentiation stages (FG and PP1) was detected at PE-PP1 common enhancers, but was depleted at the expansion-dependent PE-not-PP1 class of elements. Moreover, the VFG decommissioned enhancers VFG^OFF^-in-DE-PP1 and VFG^TR^-in-PP1 elements, which are incompletely silenced in directed differentiation, were still occupied by HHEX during these stages of directed differentiation. As a result, it appears that all enhancer classes defined here and represented in the directed differentiation dataset are occupied by HHEX, including those normally decommissioned during VFG expansion. Perhaps the binding of HHEX at these elements in directed differentiation prevents their decommissioning during rapid directed differentiation.

## Discussion

A portion of the pancreas comprising the uncinate process, in addition to the liver and gall bladder, is derived from the ventral foregut region of the developing embryo beginning at embryonic day 8.5 in mouse or at Carnegie stage 10 (25–27 days post coitum) in human^18,43^. On the basis of gene expression and differentiation competence, hESC-derived EP cells were found to recapitulate ventral foregut. While prior studies have shown that VFG expansion can produce functional pancreatic endocrine cells^15^, here we demonstrate that this is a direct consequence of time in VFG culture. In vivo, pancreas development begins from two locations, the dorsal and ventral foregut, promoting organ development via distinct signalling. Dorsal pancreas is induced by factors derived from the notochord and dorsal aorta (retinoic acid (RA), activin and FGF2) (ref. ^44^), while the ventral pancreas differentiates in the absence of signals driving hepatic specification (FGF2 produced by the cardiac mesoderm and BMP4 originating in the septum transversum)^45^. Ventral foregut embryonic explants therefore default to pancreatic differentiation in the absence of exogenous signalling^5^. However, in vivo, VFG progenitors and their descendants retain multipotency up to E11.5 in mouse where the cell cycle time has been estimated to be between 17.3 h and 26.6 h (ref. ^7^). As these progenitor cells are located close to both the cardiac mesoderm (FGF source) and septum transversum mesenchyme (BMP source), both components in VFG culture medium, these founder populations may persist via self-renewing cell division in vivo exploiting their proliferation to ensure efficient onwards differentiation.

An increasing set of TFs have the ability to bind DNA in chromatin and to destabilize nucleosomes. These pioneer factors include the FOXA proteins identified here as important for VFG priming. FOXA proteins are associated with enhancer priming during foregut development^45^ and associate with mitotic chromatin^46^. Yet, FOXA1 is not required for directed differentiation to pancreatic endoderm in vitro. While we have not shown a direct relationship between the cell cycle and enhancer priming by FOXA proteins, the major variable in our experiments is the amount of time in VFG culture and we cannot formerly exclude the influence of prolonged culture in these conditions on the enhancer network. However, it is possible that FOXA1 pioneer activity in VFG culture depends on proliferation, leading to a progressive equilibration of the enhancer network, involving both commissioning and decommissioning. Although pioneer factors are known to recognize their sites in chromatin, they may have an enhanced ability to bind their sites before the full restoration of heterochromatic marks following replication and then remain at these positions through mitosis. The hESC-directed differentiation protocol that comes closest to reproducing the proliferative nature of early ventral foregut is the one instance where a role for FOXA1 was previously suggested^47^. HHEX is also associated with enhancer priming in VFGs and can influence the stability of FOXA1 binding at the PDX1 enhancer. As HHEX physically interacts with FOXA1 in both gut tube and pancreatic progenitor stages of directed hESC differentiation^42^, it is possible that HHEX could act together with FOXA1 to enhance the stability of binding to targets in mitotic chromatin.

While we are not aware of many progenitor culture systems where the impact of proliferation on differentiation has been explored, the transition into expanded primed pluripotent cells alters the type of endoderm induced by the same cytokines^48^. It is intriguing to hypothesize that the reconfiguration of the enhancer network during the transition from naïve to primed pluripotency^49^ may also involve proliferation as cells at gastrulation stages proliferate rapidly with a cell cycle as short as 5 h as measured in rodents^50,51^. Moreover, in both naïve and primed pluripotency, the binding of pluripotency TFs to differentiation specific genes determines how these enhancers will respond to signalling and whether differentiating cells retain plasticity^52,53^, suggesting that TFs function to set the enhancer network for lineage specific progenitors to respond to signalling. In addition to preparing enhancers for later activation, we also found that enhancer decommissioning exploits expansion, perhaps as a result of going through multiple rounds of replication in the absence of specific TFs that protect these enhancers from nucleosome occlusion following replication. In VFGs, these decommissioned elements contain motifs for GATA factors, with GATA4 and GATA6 being downregulated in the early stages of VFG culture. While FOXA1 can bind mitotic chromatin, GATA factors are only partially retained^54^, suggesting that expansion could provide FOXA proteins with a competitive advantage. In this way, expansion not only primes differentiation, but shields the later developing endoderm from the lingering action of early endoderm enhancers.

We observe that the proliferation or expansion of lineage-restricted progenitors may be essential for high-efficiency later differentiation. Proliferation is therefore not just about producing sufficient numbers of cells, but fine-tuning the response of these cells to upcoming differentiation cues. Progenitor cell expansion can also equalize the differentiation efficiency of poorly performing hESCs^16,55,56^, suggesting that the lineage potential of different pluripotent cell lines may be determined by the extent they proliferate in differentiation. Moreover, as proliferation and growth are a hallmark of later foetal development, additional expansion steps could enhance the efficiency with which more mature organ-specific cell types can be obtained from human pluripotent cells.

## Methods

### Experimental design

#### Maintenance of hESC

Undifferentiated hESCs H9 (WA09, WiCell) were maintained on tissue culture plates pre-coated with 0.1% gelatine with irradiated C57BL6 mouse embryonic fibroblast feeder cells (MEFs) (25,000 cells cm^−2^) in H9 ESC medium: Dulbecco’s modified Eagle medium (DMEM)/F12 GlutaMAX medium (Thermo Fisher Scientific, 10565018) supplemented with KnockOut Serum Replacement (Thermo Fisher Scientific, 10828010), MEM Non-Essential Amino Acids (Thermo Fisher Scientific, 11140050), β-mercaptoethanol (Thermo Fisher Scientific, 21985023) and 10 ng ml^−1^ FGF2 (Peprotech, 100-18B). Cells were passaged as clusters with collagenase IV (Thermo Fisher Scientific, 17104019) when reaching approximately 70% confluence and maintained in 20% O_2_/5% CO_2_/37 °C. Undifferentiated ESC HUES4 wild-type (WT) and PDXeG clone 170-3 (ref. ^22^) were adapted and maintained in Defined Culture System (DEF-CS) (Takara, Y30017). When reaching approximately 80% confluence, cells were dissociated with TrypLE (Thermo Fisher Scientific, 12604013) and counted with the automated NucleoCounter NC-200 cell counter (Chemometec). Cells were re-plated at a density of 40,000 cells cm^−2^ and maintained in 20% O_2_/5% CO_2_/37 °C. All hESC lines were routinely screened for mycoplasma, and all were negative. All cell lines were approved for use in this project by De Videnskabsetiske Komiteer, Region Hovedstadenunder number H-4-2013-057 and H-21043866.

#### Transient differentiation of ADE cells

Transient ADE cells were generated from WT H9 and HUES4 ESCs, as well as HUES4 PDX1-eGFP reporter (PDXeG clone 170-3) ESC cell line^22^ as described in Cheng et al.^15^. In brief, ESC cells at 70–80% confluence were collected with Accutase (Thermo Fisher Scientific, 00455556), re-plated at a density of 50,000 cells cm^−2^ on polystyrene cell culture plates (Corning, 353047) pre-coated with undiluted growth factor reduced (GFR) Matrigel (Corning, 354230), cultured in either H9 ESC or DEF-CS medium for 48 h with 10 µM ROCK inhibitor Y-27632 (STEMCELL Technologies, 72302) for the first 24 h and maintained in 20% O_2_/5% CO_2_/37 °C. The ESC clusters were used to generate transient ADE cells in three-dimensional differentiation under hypoxic conditions (5% O_2_/5% CO_2_/37 °C) for 5 days. On day 1, the cell clusters were cultured in RPMI 1640 GlutaMAX (Thermo Fisher Scientific, 61870036) with 10% Serum-Free Differentiation (SFD) medium^57^ supplemented with Activin A (100 ng ml^−1^) (Peprotech, 120-14 P), CHIR99021 (3 µM) (Tocris, 4423) and 4.5 × 10^−4 ^M monothioglycerol (Sigma-Aldrich, M6145). On day 2, the medium was changed to RPMI 1640 GlutaMAX supplemented with Activin A (100 ng ml^−1^), BMP4 (0.5 ng ml^−1^) (Peprotech, 120-05ET), FGF2 (10 ng ml^−1^), VEGF (10 ng ml^−1^) (Peprotech, 100-20), 0.5 mM ascorbic acid (Sigma-Aldrich, A92902) and 4.5 × 10^−4^ M monothioglycerol. The same medium was applied at day 3. At day 4, differentiation medium was changed to SFD medium supplemented with Activin A (100 ng ml^−1^), BMP4 (0.5 ng ml^−1^), FGF2 (10 ng ml^−1^), VEGF (10 ng ml^−1^), 0.5 mM ascorbic acid and 4.5 × 10^−4^ M monothioglycerol.

#### Generation and expansion of VFG

EP/VFG expansion was performed as described^15^ with minor modifications. In brief, day-5 transient ADE clusters were dissociated with 1 volume of trypsin–EDTA (0.25%) (Thermo Fisher Scientific, 25200056) for 5 min at 37 °C and the enzyme then inactivated with 0.5 volume of foetal bovine serum (FBS) (Sigma-Aldrich, F4135). Single-cell suspensions were obtained by repeatedly washing with 10 volumes of ice-cold washing buffer, which contains 3% FBS in phosphate-buffered saline without calcium and magnesium (PBS−/−) (Thermo Fisher Scientific, 10010023). Single cells were incubated with 1:100 CD184-PEcy7 (BD Biosciences, 560669) and CD117-APC (BD Biosciences, 561118) for 45 min at 4 °C and stained with DAPI (Thermo Fisher Scientific, D3571) to exclude dead cells. CD184-CD117 double-positive cells were sorted into SFD medium with 1:100 penicillin–streptomycin (Thermo Fisher Scientific, 15140122) by fluorescence-activated cell sorting (FACS) on an SH800 (SONY SH800 Software). Sorted cells were re-plated at a density of 20,000–30,000 cells cm^−2^ on polystyrene cell culture plates pre-coated with GFR-Matrigel and pre-seeded with low-density (8,000 cells cm^−2^) irradiated DR4 MEFs (ATCC, SCRC-1045). Cells were cultured in complete EP/VFG medium (SFD medium supplemented with BMP4 (50 ng ml^−1^), FGF2 (10 ng ml^−1^), VEGF (10 ng ml^−1^), EGF (10 ng ml^−1^) (Peprotech, AF-100-15), 0.5 mM ascorbic acid and 4.5 × 10^−4^ M monothioglycerol) and maintained under hypoxic conditions (5% O_2_/5% CO_2_/37 °C).

Medium was changed every other day until cells reached confluence, at 80,000–120,000 cells cm^−2^. When VFG cells reached approximately 100 μm in diameter, they were passaged by dissociation using 1 volume of trypsin–EDTA (0.25%) for 5 min at 37 °C, detached from the plate using a cell scraper and then supplemented with 0.5 volume of FBS for enzyme inactivation. Single-cell suspension was obtained by repeatedly washing with 10 volumes of ice-cold washing buffer. VFG single cells were re-plated on the pre-coated GFR-Matrigel with feeders at 15,000–20,000 cells cm^−2^. Antibody information is listed in Supplementary Table 8.

#### Single-cell preparation for RNA-seq and index sorting

Dissociated ADE and VFG single cells with treatments (mock, BMP4 withdrawal and BMP4 withdrawal plus FGF2 stimulation) were incubated with 1:100 CD184-PEcy7 and CD117-APC for 45 mi at 4 °C, and cells were stained with DAPI to exclude dead cells. The single cells from BMP4 withdrawal plus FGF2 stimulated VFG culture were incubated only with 1:100 CD117-APC in a similar condition to that described above. Cells were sorted using a BD FACS Aria III (FACSDiva) with a 100 µm nozzle and 20 psi sheath pressure. Forward scatter (FSC) and side scatter (SSC) were used to define a homogeneous population. FSC-H/FSC-W gates were used to exclude doublets, and dead cells were excluded on the basis of DAPI inclusion. The boundary between positive and negative populations was set on the basis of a negative population of unstained cells. Sorting speed was kept at 100–300 events s^−1^ to eliminate sorting two or more cells into one well. Single-cell sorting was verified colourimetrically on the basis of a previously described protocol^58^. Cells were sorted directly into lysis buffer containing the first RT primer and RNase inhibitor, immediately frozen and later processed by the MARS-seq1 protocol as described previously^59^. All single-cell RNA-seq libraries were sequenced using Illumina NextSeq 500 at a median sequencing depth of 225,000 reads per single cell. Antibody information is listed in Supplementary Table 8.

#### Immuno-histochemical analysis

Medium was removed completely, and Matrigel-dome-containing 3D clusters were gently mixed with fresh undiluted Matrigel 1:1 and transferred to eight-well μ-slides (Ibidi, 80826) wells (20 µl cm^−2^ well) for whole-mount immunostaining. When the Matrigel was solidified at 37 °C, room-temperature 4% paraformaldehyde (Sigma-Aldrich, 158127) was added and cultures were fixed at room temperature for 10 min, blocked and permeabilized with 2% donkey serum (Jackson Immuno Research, 017-000-121), 0.3 % Triton X-100 (Sigma-Aldrich, X100) and 0.1 % BSA (Sigma-Aldrich, A7906) in PBS−/− for 1 h at room temperature. Primary antibodies were incubated with 3% FBS in PBS−/− overnight at 4 °C, subsequently incubated with the appropriate secondary antibody (Alexa Fluor, Molecular Probes) and DAPI at room temperature for 1 h. Antibody information is listed in Supplementary Table 8. Brightfield and fluorescent imaging were done using a Leica SP8 confocal microscope with Las X software (3.5.7.23225) and processed in Imaris 9.6.

#### EdU labelling and apoptosis assay

Cells were incubated with 10 µM EdU (Click-iT EdU) (Thermo Fisher Scientific, C10634) in medium for 4 h at 5% O_2_/5% CO_2_/37 °C. The 3D clusters were prepared for whole-mount immunostaining as described above. Dissociated cells were collected for flow cytometry as described above. Permeabilization, blocking and Click-iT reaction for EdU detection were performed according to the manufacturer’s instructions. Immunostaining of EdU-labelled 3D clusters were performed with antibodies supplied with the kit and with DAPI (1 μg ml^−1^) for nuclear staining. Flow cytometry of EdU-labelled dissociated cells was performed with DAPI (10 μg ml^−1^) staining cells for DNA content. Cell apoptosis was measured by Annexin V Conjugates for Apoptosis Detection kit (Thermo Fisher Scientific, A13202) according to the manufacturer’s instructions.

#### Flow cytometry

For surface marker staining, dissociated cells were incubated with conjugated antibodies for 1 h at 4 °C and were stained with DAPI (1 μg ml^−1^) to exclude dead cells. For intracellular staining, cells were stained with Ghost Dye 450 (TONBO Biosciences, 13-0868) before 4% paraformaldehyde fixation to stain dead cells. Fixed cells were permeabilized in PBS with 5% donkey serum and 0.3% Triton X-100 for 30 min at room temperature. Cells were incubated with primary antibodies in 1× PBS−/− with 5% donkey serum and 0.1% Triton X-100 overnight at 4 °C. The following day, cells were washed twice in 1× PBS and unconjugated antibodies were further incubated with secondary antibodies (Alexa Fluor conjugates) for 2 h. Antibody sources and concentrations are indicated in Supplementary Table 8. Cells were analysed using an LSR Fortessa (BD Bioscience) or FACS sorted by SH800 (SONY SH800 Software). All data were analysed with FCS Express 6 software (BD Biosciences). Antibody information is listed in Supplementary Table 8.

#### Generation of PDX1-eGFP-positive and PDX1-eGFP-negative cells with minimal cytokine sets for pancreatic spheroid and hepatic organoid expansion

PDX1-eGFP reporter VFG cells passage 6 was plated at 25,000 cells cm^−2^ on polystyrene cell culture plates pre-coated with undiluted GFR-Matrigel and pre-seeded with 8 × 10^3^ cells cm^−2^ MEFs. The cells were cultured in BMP4 withdrawal medium (SFD medium supplemented with FGF2 (10 ng ml^−1^), VEGF (10 ng ml^−1^), EGF (10 ng ml^−1^), 0.5 mM ascorbic acid and 4.5 × 10^−4^ M monothioglycerol) and maintained under hypoxic conditions (5% O_2_/5% CO_2_/37 °C) for 5 days with medium changing every other day. For generating PDX1-eGFP-positive and PDX1-eGFP-negative fractions, cells were further differentiated in DMEM high-glucose GlutaMAX Supplement (Thermo Fisher Scientific, 10566016) with 1% vol/vol B27 supplement (Thermo Fisher Scientific, 17504044), 50 ng ml^−1^ FGF2, FGF7 (Peprotech, 100-19) or FGF10 (Peprotech, 100-26) for 5 days with medium changed every day. Both BMP4 withdrawal and FGF stimulation were performed under hypoxic conditions (5% O_2_/5% CO_2_/37 °C).

The single PDX1-eGFP-positive and PDX1-eGFP-negative cells generated from the BMP4 withdrawal and FGF10-stimulated VFG culture were sorted by FACS using a SH800. GFP^+^ cells were expanded as pancreatic spheroids and GFP^–^ cells as hepatic organoids according to the described protocols^23,24^, except that the cultures were maintained under hypoxic conditions (5% O_2_/5% CO_2_/37 °C).

### Pancreatic differentiation

VFG cells at passages 6–8 were plated at 25,000 cells cm^−2^ on polystyrene cell culture plates pre-coated with undiluted GFR-Matrigel and pre-seeded with 8,000 cells cm^−2^ MEFs in the VFG medium. Day-5 expanding VFG cells were used for pancreatic differentiations under hypoxic conditions (5% O_2_/5% CO_2_/37 °C) according to protocols described as below:

For the protocol adapted from Ameri et al.^22^, day-5 expanding VFG cells were treated with DMEM high-glucose GlutaMAX Supplement with 1% vol/vol B27 supplement as basal medium throughout the differentiation and were supplemented with 2 µM RA (Sigma-Aldrich, R2625) for 3 days; then with 64 ng ml^−1^ FGF2 and 50 ng ml^−1^ hNOGGIN (R&D Systems, 6057-NG-100/CF) for 3 days; and finally with 64 ng ml^−1^ FGF2, 50 ng ml^−1^ hNOGGIN and 0.5 μM TPB (PKC activator) (Merck Millipore, 565740) for 3 days, with the medium changed every day.

For the protocol adapted from Rezania et al.^12^, day-5 expanding VFG cells were exposed to MCDB 131 basal medium (Thermo Fisher Scientific, 10372019) throughout differentiation and supplemented with 1.5 g l^−1^ sodium bicarbonate (Thermo Fisher Scientific, 25080094), 1× Glutamax Supplement (Thermo Fisher Scientific, 35050061), 10 mM d-(+)-glucose (Thermo Fisher Scientific, G8270) 0.5% BSA, 0.25 mM ascorbic acid and 50 ng ml^−1^ FGF7 for 2 days; and then with 2.5 g l^−1^ sodium bicarbonate, 1× Glutamax Supplement, 10 mM glucose, 2% BSA, 0.25 mM ascorbic acid, 1:200 insulin–transferrin–selenium–ethanolamine (ITS-X) (Thermo Fisher Scientific, 51500056), 50 ng ml^−1^ FGF7, 1 µM RA, 0.25 µM SANT-1 (Sigma-Aldrich, S4572), 100 nM LDN193189 (Tocris, 6053) and 80 nM TPB (EMD Millipore) for 2 days; and finally with 2.5 g l^−1^ sodium bicarbonate, 1× Glutamax, 10 mM glucose, 2% BSA, 0.25 mM ascorbic acid, 1:200 ITS-X, 2 ng ml^−1^ FGF7, 0.1 µM RA, 0.25 µM SANT-1, 200 nM LDN193189 and 40 nM TPB for 3 days.

For the protocol adapted from Nostro et al.^10^, day-5 expanding VFG cells were fed SFD medium supplemented with 50 ng ml^−1^ of FGF10, 3 ng ml^−1^ mouse WNT3A (R&D Systems, 1324-WN-010/CF) and 0.75 μM dorsomorphin (Sigma-Aldrich, P5499) for 3 days with the medium changed every day. Medium was then changed to DMEM high-glucose GlutaMAX Supplement with 1% vol/vol B27 supplement, 50 ng ml^−1^ FGF10, 50 ng ml^−1^ hNOGGIN, 50 μg ml^−1^ ascorbic acid and 2 µM RA, with 0.25 μM KAAD-cyclopamine (Sigma-Aldrich, 239804) for 1 day. Finally, medium was changed to DMEM high-glucose GlutaMAX Supplement with 1% vol/vol B27 supplement, 50 ng ml^−1^ hNOGGIN, 50 ng ml^−1^ EGF, 10 mM nicotinamide (Sigma-Aldrich, N0636) and 50 μg ml^−1^ ascorbic acid for 4 days with the medium changed every day.

The protocol adapted from Nostro et al.^10^ was used to assess efficiency of pancreatic differentiation in a directed protocol from ADE cells, VFGp3, VFGp6 and VFGp12 cells generated from the PDX-eGFP reporter. Day-5 transient ADE cells were generated as described previously and directly used for differentiation. Differentiation of WT H9 and HUES4 VFGp3 and VFGp6 cells to pancreatic beta-like cells were performed as reported^15,55^ with modifications during endocrine differentiation. In brief, day -13 differentiating VFG cells were re-aggregated following treatment with 1 ml Corning Cell Recovery Solution (Sigma-Aldrich, CLS354270) and cultured on the membrane surface of Millicell insert (Millipore, PICM03050) in the same medium described in Tiya et al.^55^.

#### Hepatic and intestinal differentiations

Hepatic and intestinal differentiations were started from day-5 expanding VFG cells according to the protocols described in Cheng et al.^15^.

#### Total mRNA purification, reverse transcription and qPCR analysis

Two hundred thousand cells were washed in 1× PBS twice, lysed in RLT buffer (RNeasy Micro kit) (Qiagen, 74004) containing 1% β-mercaptoethanol (Sigma-Aldrich, M6250) and stored at −80 °C until processing. Total mRNA was isolated using the RNeasy Micro kit according to the manufacturers’ instructions and digested with RNase-free DNase I, (Qiagen, 79254) to remove genomic DNA. First-strand complementary DNA synthesis was performed with SuperScript III First-Strand Synthesis System (Thermo Fisher Scientific, 18080051) using random hexamers (Thermo Fisher Scientific, N8080127) and amplified using SYBR Green PCR Master Mix (Thermo Fisher Scientific, 4309155). PCR primers were designed using Primer3Plus^60^ and validated for efficiency ranging between 95% and 100%. Primer sequences used in quantitative reverse transcription PCR (RT–qPCR) are listed in Supplementary Table 9. StepOnePLUS Real-Time PCR System (Thermo Fisher Scientific) was used for RT–qPCR in 96-well plate format. Expression values for each gene were normalized against *ACTB*, using the delta–delta CT method.

#### Sample preparation for bulk RNA-seq

Total mRNA amount and RNA integrity were assessed using a Fragment Analyzer (AATI). Ribosomal RNA was removed from samples using the NEBNext Poly(A) mRNA Magnetic Isolation Module (NEB, E7490L). Sequencing libraries were prepared from 100 ng of purified total mRNA using NEBNext Ultra II RNA Library Prep Kit for Illumina (NEB, E7770L) according to the manufacturer’s instructions. RNA-seq libraries were sequenced for 75 cycles in single-end mode on NextSeq 500 platform (Illumina, FC-404-2005).

#### Sample preparation for ATAC-seq

Dissociated single cells were washed with ice-cold PBS−/− and pelleted at 500*g* for 10 min at 4 °C. Fifty-thousand cells were taken from a diluted stock in PBS buffer to prepare ATAC-seq libraries as described in Buenrostro et al.^61^ with slight modifications. Nuclei were prepared by resuspending the cells in 100 µl ice-cold ATAC lysis buffer (10 mM Tris–HCl pH 7.4, 10 mM NaCl, 3 mM MgCl_2_ and 0.1% NP40) followed by incubation on ice for 15 min while mixing every 5 min. Nuclei were then collected by centrifuging at 1,000*g* for 10 min at 4 °C, and the pellet was resuspended in 50 µl transposition buffer (10 mM Tris pH 8, 5 mM MgCl_2_ and 10% dimethylformamide). Tagmentation was performed by adding 2.5 µl Tn5 transposase (Illumina, 20034197) and incubating at 37 °C while shaking in a thermomixer set at 1,000 rpm. Tagmentation reactions were stopped and purified with MinElute PCR Purification Kit (Qiagen, 28004) and tagmented DNA eluted in 10 µl elution buffer (10 mM Tris pH 8.0). A 50 µl PCR reaction was assembled containing 10 µl of tagmented DNA, 25 µl NEBNext High-Fidelity PCR Mix (NEB, M0541S*)*, 5 µl of SYBR Green (Invitrogen, S7563) and index primers at 2 µM concentration. Ten microlitres of each PCR reaction was used to decide the optimum number of PCR cycles required with following conditions: 5 min at 72 °C; 30 s at 98 °C; and 20 cycles of 10 s at 98 °C, 30 s at 63 °C and 60 s at 72 °C. The reaction was monitored in a LightCycler-480 qPCR (Roche), and the number of cycles required was deduced from the amplification curve. The remaining PCR reaction was then subjected to this number of PCR cycles. The PCR reaction was purified with an equal volume of AMPure XP beads (Beckman, A63880) following manufacturer’s protocol and was eluted in 20 µl Tris pH 7.8. Libraries were quantified with Qubit dsDNA High-sensitivity Assay (Invitrogen, Q32851), and fragment profiles were checked using Bioanalyzer High Sensitivity assay (Agilent) or Fragment Analyzer (AATI). Samples that showed nucleosomal bands were sequenced for 75–150 cycles in paired-end mode on an Illumina HiSeq-2000 platform or NextSeq 500.

#### Generation of shRNA KD VFG cell lines

shRNAs targeting *HHEX*, *FOXA1* and *FOXA2* transcripts were designed using RNAi consortium (TRC) GPP Web Portal (Broad Institute) (https://portals.broadinstitute.org/gpp/public) (for *HHEX*, *FOXA1* and *FOXA2* shRNA sequences, see Supplementary Table 9). A vector delivering a scrambled sequence was used as control (for scrambled shRNA sequence, see Supplementary Table 9). All shRNA sequences were cloned into a lentiviral vector (pL-U6-sgRNA-SFFV-Puro-P2A-EGFP), a gift from Kristian Helin (Addgene, 175037) (ref. ^62^), using *BsmBI* sites. HEK293FT packaging cells were co-transfected with the pL-U6-sgRNA-SFFV-Puro-P2A-EGFP carrying individual shRNAs and pAX8 and pCMV-VSV using Lipofectamine 2000 supplemented with polyethylenimine (Sigma-Aldrich, 408727) according to standard protocols. SFD medium carrying lentivirus produced from HEK293FT cells (48 h post-transduction) was applied 1:1 with fresh VFG expansion medium to one 12-well plate of day 2 VFG cell culture (passaged at 25,000 cells cm^−2^ at day 0). Transduction was performed in presence of 1:1,000 polybrene infection/transfection reagent (Merck Millipore, TR-1003-G) at 8 µg ml^−1^. Forty-eight hours after transduction with the sgRNA-encoding lentiviral plasmids, the VFG cells were selected and maintained at 0.25 μg ml^−1^ puromycin in standard VFG condition.

#### ChIP–qPCR

ChIP was carried out using the True MicroChIP kit (Diagenode, C01010132) with modifications. One-hundred-thousand sorted CD184-CD117 double-positive cells ADE, VFGp3 and VFGp6 cells; or shRNAs (scrambled, FOXA1, FOXA2 or HHEX) KD VFGp6 cells were fixed in 1% formaldehyde (Thermo Fisher Scientific, 28906) in ADE or VFG medium for 10 min at room temperature followed by a 5 min quench with glycine (in True MicroChIP kit, Diagenode) at room temperature. Cells were lysed and immunoprecipitation performed using the True MicroChIP kit (Diagenode, AB-002-0016) with the following modifications. Up to 100,000 cells were sonicated in one lysate and split into 50,000 equivalents after sonication. Samples were lysed using 50 µl of buffer tL1 and incubated for 5 min on ice. One-hundred-fifty microlitres of Hank’s buffered salt solution with 1× protease inhibitor cocktail (in True MicroChIP kit, Diagenode) was added, and the lysate was sonicated in 0.65 ml Bioruptor Pico Microtubes (Diagenode, C30010020). Chromatin was sheared using a Bioruptor Pico (Diagenode) with ten cycles (30 s on, 30 s off). Sonicate was aliquoted in 100 µl (for 50,000 cells), and an equivalent volume of complete ChIP buffer tC1 was added. For immunoprecipitation, the following antibodies and amounts of antibody were used for the 50,000-cell ChIP: 2 µg of FOXA1 (1:50) (Abcam, ab170933), 2 µg of H3K4me1 (1:50) (Abcam, ab8895), 2 µg of H3K27ac (1:50) (Abcam, ab4279) and 2 µg of HHEX (1:100) (R&D, MAB83771). Immunoprecipitation and washes were as described in the True MicroChIP protocol, then purified by phenol chloroform extraction and ethanol precipitation. The pull-down DNA was eluted in 100 µl elution buffer and qPCR was performed as described in the True MicroChIP protocol for different genomic loci. Enrichment was calculated as percentage of input. Antibody information is listed in Supplementary Table 8. The primer sequences used in ChIP–PCR are listed in Supplementary Table 9.

#### In vitro scRNA-seq analysis

Sequences were mapped to the hg38 assembly of the human genome, de-multiplexed and filtered as previously described^59,63^ extracting a set of unique molecular identifiers (UMIs) that define distinct transcripts in single cells for further processing. We estimated the level of spurious UMIs in the data using statistics on empty MARS-seq wells as previously described^59^. Mapping of reads was done using HISAT (version 0.1.6) (ref. ^64^). Reads with multiple mapping positions were excluded. Reads were associated with genes if they mapped to an exon. Raw counts were further analysed using Seurat (4.0.1) (ref. ^65^) (https://satijalab.org/seurat/). Cells were filtered with the following thresholds (lower bound: 2,000 UMIs; 550 genes and upper bound: 35,000 UMIs; 4,950 genes). Additionally, cells with more than 20% of mitochondria content were removed. In Extended Data Fig. 1a, we subset ADE and VFG cells (505 cells). Raw counts were further normalized, log-transformed and scaled using NormalizeData and ScaleData, respectively. PCA was computed on 2,000 highly variable genes without cell cycle regression. The dataset was clustered using Louvain with 0.7 resolution followed by uniform manifold approximation and projection dimension reduction on top 20 PCs. In Extended Data Fig. 2d, we subset for treated and withdrawal cells (562 cells). We follow the same steps above adjusting only clustering resolution set to 0.5. Detailed analyses can be found at https://github.com/brickmanlab/wong-et-al-2022/.

#### In vivo scRNA-seq re-analysis

The Li et al.^20^ dataset HRA000280 was downloaded from Genome Sequence Archive. Cells with low quality and mitochondrial content higher than 20% were filtered out (lower bound: 3,000 genes and upper bound: 9,000 genes; 400,000 UMIs). Additionally, cells labelled as ‘poor quality’ were also discarded. We followed the same pre-processing steps as mentioned above without clustering. We subsetted the final dataset for hMG, hHG, hFG and hAL population.

#### CAT

We used CAT to determine similarity between clusters from in vivo and in vitro studies. CAT calculates mean gene expression of randomly sampled cells with replacement for each cluster 1,000 times. Euclidian distance is measured between all pairs of clusters. A small distance represents high similarity. A detailed explanation of the method can be found in Rothova et al.^19^.

#### Analysis of bulk RNA-seq data

Fastq files from bulk RNA-seq samples were aligned to the hg38/GRCh38 genome using STAR v2.5.3a^66^. Transcript expression levels were estimated with the quantMode GeneCounts option and GRCh38p10.v27 annotations. FastQC v0.11.7 (http://www.bioinformatics.babraham.ac.uk/projects/fastqc) was used for quality control metrics and multiqc v1.7 (ref. ^67^) for reporting. Data analysis was then performed with R/Bioconductor^68^ (https://www.R-project.org). Normalization was performed with DEseq2 (v1.24.0) (ref. ^69^). The Lee et al.^35^ dataset was retrieved from NCBI GEO (GSE114102) and analysed as above. Differential gene expression was assessed using DESeq2 (R package version 1.32.0). *Z*-scoring was calculated as previously described for each dataset separately. Gene set enrichment analysis was performed by Webgestalt (http://www.webgestalt.org) (log_2_FC between VFGp3 and VFGp6) for Gene Ontology Biological Process (GO-BP) with false discovery rate <0.05.

#### Processing of ATAC-seq datasets

The quality of the sequencing reads was assessed with FastQC (https://www.bioinformatics.babraham.ac.uk/projects/fastqc/) followed by trimming of poor-quality base calls and adaptor sequences with cutadapt^70^. Read pairs were then aligned to the hg19 reference genome using bowtie2 (ref. ^71^) with the following parameters: bowtie2–no-discordant–no-mixed–no-unal–very-sensitive -X 2000. Samtools^72^ was used for sorting alignments and format conversions. Alignments from PCR duplicates were removed using Picard (http://broadinstitute.github.io/picard/). Alignments were then converted into BED format using bedtools^73^. The 5′ ends of the reads were offset by +4 bases for the reads on Watson strand and by −5 bases for the reads on Crick strand, to reflect the exact location of Tn5 insertion site. Single-base genome-wide coverage was computed using a 30 bp fragment centred at the Tn5 insertion site in BigWig format. We called peaks using Macs2 (ref. ^74^) with the following parameters: macs2 callpeak–nomodel–extsize 150–shift -75 -g ‘hs’ -p 0.01. For each condition, data from two biological replicates were used to create a set of highly reproducible peaks using irreproducible discovery rate (≤0.05, ref. ^75^). Deeptools^76^ was employed to compute Pearson’s correlation among the conditions/replicates and for PCA plots. Bedtools intersect command was used to find overlapping or unique (with parameter ‘-v’) enhancer positions (bed format) between two conditions in question (Fig. 3b).

#### Detection of differential chromatin accessibility and temporal dynamics of enhancers from ATAC-seq data

A consensus set of ATAC-seq peaks was created using reproducible peaks from all five stages of differentiation. Next, we computed normalized read coverage (RPKM) for the consensus peak set in all stages. General linear modelling was applied to the normalized counts from the step above to detect changes in chromatin accessibility across the stages and in both directions. We used the following parameters for differential accessibility: log_2_FC > 2 or log_2_FC < 2 at adjusted *P* value <0.005 (time-course sequencing (TC-seq), ref. ^30^). We then defined stage-specific peaks using *c*-means clustering of the dynamic peak-set from the step above. We called eight clusters that gave a functionally relevant pattern along the timeline of differentiation. Some clusters were merged, as they were too similar to be dealt with separately. This led to formation of the six groups of dynamic enhancers (Fig. 3c, right). RPKM-normalized BigWig tracks from merged replicates were used to plot heat maps in deeptools^76^. For locus-specific visualizations, we used the UCSC Genome Browser (http://genome.ucsc.edu, ref. ^77^) to load BigWig tracks.

#### Enrichment scoring of defined ATAC-clusters from the mapped gene sets that are up- or downregulated at the PE stage compared with VFGp6

ATAC-seq peaks were assigned to genes using GREAT^78^ with the setting of single nearest gene within 25 or 200 kb (Supplementary Table 1b). The enrichment of gene-annotated ATAC clusters in differential expression gene sets was calculated by log_2_ ratio between number of observed overlaps and number of expected overlaps from the dataset. We compared the impact of very low levels of background gene expression noise (those genes not reaching more than 100 or 1,000 reads in a particular sample, baseMean 100 or 1,000) on these gene sets (Supplementary Table 1d–g). While filtering out gene expression noise reduces the size of the gene set, it can be expanded by considering enhancers located within 200 kb of a target gene.

#### Motif analysis from ATAC-clusters

Enrichment of known and de novo TF binding motifs was calculated with the HOMER v4.11.1 suite^79^ using the findMotifsGenome function with default parameters.

#### Hierarchical *k*-means clustering of expression patterns of genes annotated to ATAC-peaks clusters

Bulk RNA-seq gene expression levels were normalized using DESeq2 R package version 1.32.0 (ref. ^69^). The mean of normalized expression was calculated for each condition and transformed into *z*-scores. Gene expression levels were then separated into the different annotated ATAC-peaks clusters. Finally, gene expression patterns were grouped using hierarchical clustering (*k* = 10) based on Euclidian distances.

#### Mapping and analysis of H3K27ac data from human embryo samples

Pre-processing and alignment of ChIP–seq reads was as described in Gerrard et al.^37^. Single-end reads were aligned to hg19 genome assembly with bowtie 1.0.0 (parameters: -m1 –n 2 –l 28, uniquely mapped reads only). These alignments were received in compressed BAM format from European Genome-Phenome Archive (https://ega-archive.org/) under accession numbers EGAS00001003163 and EGAS0001004335. We converted the alignments to BED format and called peaks with HOMER (parameters: findPeaks -style histone) against a pooled input sample. We then used bedtools-2.30 (ref. ^73^) to select the peaks present in both replicates (bedtools intersect –f 0.50 –r –u -a rep1.bed –b rep2.bed) of most tissue types except for stomach.

Lineage-specific sets of H3K27ac regions were generated by concatenating peaks from relevant tissues as follows: ectoderm (RPE and brain), endoderm (pancreas, liver, lung and stomach) and mesoderm (heart and adrenal). To identify unique regions for each germ layer, we use bedtools intersect command, followed by sorting regions using sort option and finally merging smaller regions that are subsets of larger regions using bedtools merge command. This process ensures a unique count of peaks even if a given peak is part of a larger regulatory region. Similarly, we identified regions unique to tissue types. To map different ATAC clusters to the H3K27ac regions described above, we took regions in different enhancer classes and intersected these with different classes of H3K27ac regions from human foetal samples with bedtools intersect command. These overlaps were used in generating over-representation scores defined as observed/expected.

#### Enrichment scoring of dynamic ATAC-seq clusters with H3K27Ac regions from human embryonic tissues

The enrichment of ATAC clusters in different lineage- and tissue-specific H3K27ac groups was calculated on the basis of the ratio between number of observed overlapped regions (between ATAC and H3K27ac peaks) and number of expected overlapped regions from the datasets.

#### Analysis of HHEX ChIP–seq dataset

We aligned HHEX ChIP–seq data from Yang et al.^42^ to hg19 assembly using bowtie-1.3.1 (ref. ^80^) with default parameters and converted the alignments to HOMER tag-directory format. We created depth-normalized bigwig files using the HOMER^79^ makeUCSCfile program. ComputeMatrix (deeptools suite^76^) was used to plot the coverage centred at the midpoint of enhancer regions in different classes (Extended Data Fig. 10g). This dataset can be found on NCBI GEO under accession number GSE181480.

#### Statistical analyses and reproducibility

No statistical methods were used to pre-determine sample size. Data distribution was assumed to be normal, but this was not formally tested. The experiments were not randomized. Data collection and analysis were not performed blind to the conditions of the experiments. No data points were excluded from the analyses. Data collection was performed using Microsoft Office Excel (16.16.2). Data representation and statistical analyses were performed using GraphPad Prism. Unless mentioned otherwise, data are shown as mean ± standard error of the mean (s.e.m.) and *N* numbers refer to biologically independent replicates. Statistical significance (*P* < 0.05) was determined as indicated in figure legends using one-way analysis of variance (ANOVA) Tukey’s multiple comparison test (Figs. 1c,d, 2d and 4b,c and Extended Data Fig. 3d–f), one-way ANOVA Dunnett’s multiple comparison test (Figs. 2a,b and 6b,c and Extended Data Figs. 2h,i, 9a,c and 10a,b), unpaired two-tailed *t*-test (Fig. 2d,e), unpaired one-tailed *t*-test (Fig. 6d–f and Extended Data Fig. 10c–f) and chi-squared test (Figs. 3f,g and 4d,e and Extended Data Fig. 5d).

### Reporting summary

Further information on research design is available in the Nature Portfolio Reporting Summary linked to this article.

## Online content

Any methods, additional references, Nature Portfolio reporting summaries, source data, extended data, supplementary information, acknowledgements, peer review information; details of author contributions and competing interests; and statements of data and code availability are available at 10.1038/s41556-022-01075-8.

## Supplementary information

Reporting Summary

Supplementary Table 1 Summary of dynamic ATAC peaks and their annotated genes classified by differential expression between VFGs and PE samples.

Supplementary Table 2 Summary of VFG-specific ATAC peaks and their annotated genes classified by differential expression between VFG and PE samples.

Supplementary Table 3 H3K27ac datasets derived from different human embryonic tissues.

Supplementary Table 4 Dynamic enhancers defined by ATAC-seq clusters mapped to H3K27ac datasets from human embryonic tissues.

Supplementary Table 5 Summary of VFG expansion-specific ATAC peaks and their annotated genes classified by differential expression between VFGs and PE samples.

Supplementary Table 6 VFG expansion-specific enhancers defined by ATAC-seq clusters mapped to H3K27ac datasets from human embryonic tissues.

Supplementary Table 7 Motif enrichment results for dynamic and VFG expansion-specific enhancers defined by ATAC-seq clusters.

Supplementary Table 8 Antibodies used in this study.

Supplementary Table 9 Oligonucleotide sequences used in this study.

## Data Availability

Sequencing data generated in this study are available on NCBI GEO under the accession numbers GSE185670 (bulk RNA-seq), GSE188362 (single-cell RNA-seq) and GSE108623 (ATAC-seq). The Lee et al.^35^ dataset reanalysed here can be found at NCBI GEO under accession number GSE114102. The human embryo H3K27ac ChIP–seq data reanalysed here and based on our previous study^37^ are available on European Genome Phenome repository (EGAS00001004335 and EGAS00001003163). The ChIP-seq dataset for HHEX binding during pancreatic differentiation and reanalysed here^42^ can be found on NCBI GEO under accession number GSE181480. Processed data and gene lists from various analysis are included as supplementary tables. Source data are provided with this paper. All other data supporting the findings of this study are available from the corresponding authors on reasonable request. Cell lines and reagents generated for this study are available from the corresponding authors with a complete Materials Transfer Agreement.

## Source data

Source Data Fig. 1 Source data for Fig. 1c,d.
Source Data Fig. 2 Source data for Fig. 2a,b,d,e,g.
Source Data Fig. 3 Source data for Fig. 3d–g.
Source Data Fig. 4 Source data for Fig. 4b–e.
Source Data Fig. 5 Source data for Fig. 5a,c,d.
Source Data Fig. 6 Source data for Fig. 6b–f.
Source Data Extended Data Fig.1 Source data for Extended Data Fig. 1d,e.
Source Data Extended Data Fig.2 Source data for Extended Data Fig. 2h,i.
Source Data Extended Data Fig.3 Source data for Extended Data Fig. 3d–f.
Source Data Extended Data Fig.4 Source data for Extended Data Fig. 4e.
Source Data Extended Data Fig.5 Source data for Extended Data Fig. 5d.
Source Data Extended Data Fig.7 Source data for Extended Data Fig. 7a–d.
Source Data Extended Data Fig.9 Source data for Extended Data Fig. 9a,c.
Source Data Extended Data Fig.10 Source data for Fig. Extended Data 10a–f.

## Code availability

Code used to perform the analyses in this study is available at https://github.com/brickmanlab/wong-et-al-2022/ or from the corresponding authors upon request.
