# Supplementary material for: Expansion of ventral foregut is linked to changes in the enhancer landscape for organ-specific differentiation
Source: Nat Cell Biol. 2023 Jan 23;25(3):481–92. doi: 10.1038/s41556-022-01075-8 (PMC10014581; doi:10.1038/s41556-022-01075-8)
Supplement: Supplementary file 1 — Reporting Summary [file 41556_2022_1075_MOESM1_ESM.pdf]

nature portfolio

Corresponding author(s): Joshua M. Brickman
Wendy A. Bickmore

Last updated by author(s): Nov 28, 2022

Reporting Summary

Nature Portfolio wishes to improve the reproducibility of the work that we publish. This form provides structure for consistency and transparency in reporting. For further information on Nature Portfolio policies, see our Editorial Policies and the Editorial Policy Checklist.

Statistics

For all statistical analyses, confirm that the following items are present in the figure legend, table legend, main text, or Methods section.

n/a	Confirmed	
☐	☒	The exact sample size (*n*) for each experimental group/condition, given as a discrete number and unit of measurement
☐	☒	A statement on whether measurements were taken from distinct samples or whether the same sample was measured repeatedly
☐	☒	The statistical test(s) used AND whether they are one- or two-sided<br>*Only common tests should be described solely by name; describe more complex techniques in the Methods section.*
☒	☐	A description of all covariates tested
☐	☒	A description of any assumptions or corrections, such as tests of normality and adjustment for multiple comparisons
☐	☒	A full description of the statistical parameters including central tendency (e.g. means) or other basic estimates (e.g. regression coefficient) AND variation (e.g. standard deviation) or associated estimates of uncertainty (e.g. confidence intervals)
☐	☒	For null hypothesis testing, the test statistic (e.g. *F*, *t*, *r*) with confidence intervals, effect sizes, degrees of freedom and *P* value noted<br>*Give P values as exact values whenever suitable.*
☒	☐	For Bayesian analysis, information on the choice of priors and Markov chain Monte Carlo settings
☒	☐	For hierarchical and complex designs, identification of the appropriate level for tests and full reporting of outcomes
☒	☐	Estimates of effect sizes (e.g. Cohen's *d*, Pearson's *r*), indicating how they were calculated

Our web collection on statistics for biologists contains articles on many of the points above.

Software and code

Policy information about availability of computer code

Data collection

- BD FACSDiva™ Software version 8 was used for index sorting of cells for single cell RNA-seq and acquisition of LSR Fortessa.
- SONY SH800 Software version 1.8 was used for cell sorting.
- Las X software (3.5.7.23225) was used for taking image.
- Illumina NextSeq 500 (v4.0.1) was used for both single-cell RNA-seq, bulk RNA-seq.
- Illumina HiSeq-2000 (HCS 2.2.68) and NextSeq 500 (4.0.1) platforms were used for ATAC-seq.
- StepOnePLUS Real-Time PCR System (v2.3) for quantitative PCR Analysis.
- Roche LC480 LightCycler (1.5.1.62.SP3) for optimizing production of ATAC-seq library.
- Agilent 2100 Bioanalyzer (B.01.03) for optimizing production of ATAC-seq library.
- AATI Fragment Analyzer (v1.0.0.2) for optimizing production of RNA-seq library.

Data analysis

1. GraphPad Prism version 8 for Mac OS X
2. Microsoft Excel 16.16.2 for Mac OS X
3. FCS Express 6
4. IMARIS 9.6
5. HISAT (version 0.1.6)
6. DESeq2 (R package version 1.32.0)
7. R version 4.1.2 and following packages were used for single-cell RNA-seq analysis

abind: 1.4.5, annotate: 1.72.0, AnnotationDbi: 1.56.2, Biobase: 2.54.0, BiocGenerics: 0.40.0, BiocParallel: 1.28.3, Biostrings: 2.62.0, bit: 4.0.4, bit64: 4.0.5, bitops: 1.0.7, blob: 1.2.2, cachem: 1.0.6, callr: 3.7.0, cellranger: 1.1.0, cli: 3.1.0, cluster: 2.1.2, codetools: 0.2.18, colorspace: 2.0.2, commonR: 0.1.0, cowplot: 1.1.1, crayon: 1.4.2, data.table: 1.14.2, DBI: 1.1.2, DelayedArray: 0.20.0, deldir: 1.0.6, desc: 1.4.0, DESeq2: 1.34.0, devtools: 2.4.3, digest: 0.6.29, dplyr: 1.0.7, ellipsis: 0.3.2, evaluate: 0.14, fansi: 1.0.2, fastmap: 1.1.0, fitdistrplus: 1.1.6, fs: 1.5.2, future: 1.23.0, future.apply: 1.8.1, genefilter: 1.76.0, geneplotter: 1.72.0, generics: 0.1.1, GenomeInfoDb: 1.30.0, GenomeInfoDbData: 1.2.7, GenomicRanges: 1.46.1, ggplot2: 3.3.5, ggrepel: 0.9.1, ggridges: 0.5.3, ggsignif: 0.6.3, globals: 0.14.0, glue: 1.6.0, goftest: 1.2.3, gridExtra: 2.3,

gtable: 0.3.0, hdf5r: 1.3.5, htmltools: 0.5.2, htmlwidgets: 1.5.4, httpuv: 1.6.5, httr: 1.4.2, ica: 1.0.2, igraph: 1.2.11, IRanges: 2.28.0, irlba: 2.3.5, jsonlite: 1.7.2, KEGGREST: 1.34.0, KernSmooth: 2.23.20, knitr: 1.37, later: 1.3.0, lattice: 0.20.45, lazyeval: 0.2.2, leiden: 0.3.9, lifecycle: 1.0.1, listenv: 0.8.0, lmtest: 0.9.39, locfit: 1.5.9.4, magrittr: 2.0.1, MASS: 7.3.55, Matrix: 1.4.0, MatrixGenerics: 1.6.0, matrixStats: 0.61.0, memoise: 2.0.1, mgcv: 1.8.38, mime: 0.12, miniUI: 0.1.1.1, munsell: 0.5.0, nlme: 3.1.155, parallelly: 1.30.0, patchwork: 1.1.1, pbapply: 1.5.0, pheatmap: 1.0.12, pillar: 1.6.4, pkgbuild: 1.3.1, pkgconfig: 2.0.3, pkgload: 1.2.4, plotly: 4.10.0, plyr: 1.8.6, png: 0.1.7, polyclip: 1.10.0, prettyunits: 1.1.1, processx: 3.5.2, promises: 1.2.0.1, ps: 1.6.0, purrr: 0.3.4, R6: 2.5.1, RANN: 2.6.1, RColorBrewer: 1.1.2, Rcpp: 1.0.8, RcppAnnoy: 0.0.19, RCurl: 1.98.1.5, readxl: 1.3.1, remotes: 2.4.2, reshape2: 1.4.4, reticulate: 1.23, rlang: 0.4.12, rmarkdown: 2.11, ROCR: 1.0.11, rpart: 4.1.15, rprojroot: 2.0.2, RSQLite: 2.2.9, rstudioapi: 0.13, Rtsne: 0.15, S4Vectors: 0.32.3, scales: 1.1.1, scattermore: 0.7, sctransform: 0.3.3, sessioninfo: 1.2.2, Seurat: 4.1.0, SeuratDisk: 0.0.0.9019, SeuratObject: 4.0.4, shiny: 1.7.1, spatstat.core: 2.3.2, spatstat.data: 2.1.2, spatstat.geom: 2.3.1, spatstat.sparse: 2.1.0, spatstat.utils: 2.3.0, stringi: 1.7.6, stringr: 1.4.0, SummarizedExperiment: 1.24.0, survival: 3.2.13, tensor: 1.5, testthat: 3.1.1, tibble: 3.1.6, tidyr: 1.1.4, tidyselect: 1.1.1, usethis: 2.1.5, utf8: 1.2.2, uwot: 0.1.11, vctrs: 0.3.8, viridisLite: 0.4.0, withr: 2.4.3, xfun: 0.29, XML: 3.99.0.8, xtable: 1.8.4, XVector: 0.34.0, yaml: 2.2.1, zlibbioc: 1.40.0, zoo: 1.8.9

All custom software is deposited at GitHub
https://github.com/brickmanlab/wong-et-al-2022

For manuscripts utilizing custom algorithms or software that are central to the research but not yet described in published literature, software must be made available to editors and reviewers. We strongly encourage code deposition in a community repository (e.g. GitHub). See the Nature Portfolio guidelines for submitting code & software for further information.

Data

Policy information about availability of data

All manuscripts must include a data availability statement. This statement should provide the following information, where applicable:

- Accession codes, unique identifiers, or web links for publicly available datasets
- A description of any restrictions on data availability
- For clinical datasets or third party data, please ensure that the statement adheres to our policy

- Sequencing data generated in this study is available on NCBI GEO under the accession numbers GSE185670 (bulk RNA-seq), GSE188362 (single-cell RNA-seq), and GSE108623 (ATAC-seq).
- The Lee et al. dataset reanalyzed here can be found at NCBI GEO under the accession number GSE114102 (Lee, K. et al. FOXA2 Is Required for Enhancer Priming during Pancreatic Differentiation. Cell Reports 28, 382-393.e7 (2019)).
- The human embryo H3K27ac ChIP–seq dataset of human embryos is reanalyzed here and based on our from our previous study and is available on European Genome Phenome repository (EGAS00001004335 and EGAS00001003163) (Gerrard, D. T. et al. Dynamic changes in the epigenomic landscape regulate human organogenesis and link to developmental disorders. Nat Commun 11, 3920 (2020)).
- The ChIP-seq dataset for HHEX binding during pancreatic differentiation and reanalyzed here can be found on NCBI GEO under accession number GSE181480 (Yang, D. et al. CRISPR screening uncovers a central requirement for HHEX in pancreatic lineage commitment and plasticity restriction. Nat. Cell Biol. 24, 1064–1076 (2022)).

Field-specific reporting

Please select the one below that is the best fit for your research. If you are not sure, read the appropriate sections before making your selection.

☒ Life sciences ☐ Behavioural & social sciences ☐ Ecological, evolutionary & environmental sciences

For a reference copy of the document with all sections, see nature.com/documents/nr-reporting-summary-flat.pdf

Life sciences study design

All studies must disclose on these points even when the disclosure is negative.

Sample size	- Sample size was chosen based on previously published literature in this area (Cheng, X. et al. Self-renewing endodermal progenitor lines generated from human pluripotent stem cells. Cell Stem Cell 10, 371–384 (2012)).<br>- Biological replicates were at least N = 3 unless otherwise noted. These are clearly showed with the dot-plot overlaying the bar graph.<br>- All attempts at replication were successful.<br>- For single-cell RNA-seq, we sampled 1067 cells spread across 5 samples.
Data exclusions	- For scRNA-seq data, we excluded cells with following thresholds: minimum 2,000 UMIs; 550 genes and maximum 35,000 UMIs; 4,950 genes. Additionally, cells with higher than 20% mitochondrial content were discarded.<br>- For other experiments, no data were excluded.
Replication	Biological replicates were performed at least 3 times. The mean +/- S.E.M, and statistical analysis (ANOVA Tukey's multiple comparison test, one-way ANOVA Dunnett's multiple comparison test, unpaired two-tailed t-test, unpaired one-tailed t-test) was used to see if and how the difference between parameters are significant. Significance were presented as stars, calculated by Graph Pad software and described under Methods.
Randomization	There were no randomization as there were no clinical studies or patient participants were involved. Moreover, we included appropriate controls in all experiments, so the randomization was not required.
Blinding	The investigators were not blinded to allocation during experiments or analysis, as group allocation was clearly visible in the samples due to

Blinding | phenotypic changes.

Reporting for specific materials, systems and methods

We require information from authors about some types of materials, experimental systems and methods used in many studies. Here, indicate whether each material, system or method listed is relevant to your study. If you are not sure if a list item applies to your research, read the appropriate section before selecting a response.

Materials & experimental systems

n/a	Involved in the study
☐	☒ Antibodies
☐	☒ Eukaryotic cell lines
☒	☐ Palaeontology and archaeology
☒	☐ Animals and other organisms
☒	☐ Human research participants
☒	☐ Clinical data
☒	☐ Dual use research of concern

Methods

n/a	Involved in the study
☒	☐ ChIP-seq
☐	☒ Flow cytometry
☒	☐ MRI-based neuroimaging

Antibodies

Antibodies used

Below is a summary of commercially available antibodies used in this study: or as cited in the Materials and methods section:
FOXA2 R&D Systems AF2400 1:500
HHEX R&D Systems MAB83771 Clone#2018B 1:100
TBX3 abcam ab99302 1:500
PDX1 R&D Systems AF2419 1:500
SOX9 Millipore AB5535 1:300
AFP R&D Systems MAB1369 Clone#189506 1:500
ALB R&D Systems AF3329 1:500
NKX6-2 abcam ab58708 1:100
ROBO2 Cell Signaling Technology 45568S 1:100
GP2 MBL INTERNATIONAL D277-5 3G7-H9 1:100
INS Dako a0564 1:1000
H3K4me1 abcam ab8895 1:50
H3K27ac abcam ab4729 1:50
FOXA1 abcam ab170933 EPR10881 1:50
CD184-PECy7 BDBiosciences 560669 12G5 (RUO) 1:100
CD117-APC BDBiosciences 561118 YB5.B8 (RUO) 1:100
Alexa fluor 488 anti-goat Thermo Fisher A11055 1:800
Alexa fluor 568 anti-goat Thermo Fisher A11057 1:800
Alexa fluor 647 anti-goat Thermo Fisher A21447 1:800
Alexa fluor 488 anti-mouse Thermo Fisher A21202 1:800
Alexa fluor 568 anti-mouse Thermo Fisher A10037 1:800
Alexa fluor 488 anti-rabbit Thermo Fisher A32790 1:800
Alexa fluor 568 anti-rabbit Thermo Fisher A10042 1:800
Alexa fluor 647 anti-rabbit Thermo Fisher A32795 1:500

Validation

Antibodies were validated as noted on manufacturer's websites as following:
- FOXA2 R&D Systems AF2400 1:500; https://www.rndsystems.com/products/human-hnf-3beta-foxa2-antibody_af2400
- HHEX R&D Systems MAB83771 Clone#2018B 1:100; https://www.rndsystems.com/products/human-mouse-rat-hhex-antibody-2018b_mab83771
- TBX3 abcam ab99302 1:500; https://www.abcam.com/tbx3-antibody-ab99302.html
- PDX1 R&D Systems AF2419 1:500; https://www.rndsystems.com/products/human-pdx-1-ipf1-antibody_af2419
- SOX9 Millipore AB5535 1:300; https://www.merckmillipore.com/DK/en/product/Anti-Sox9,MM_NF-AB5535-25UG?ReferrerURL=https%3A%2F%2Fwww.google.com%2F&bd=1
- AFP R&D Systems MAB1369 Clone#189506 1:500; https://www.rndsystems.com/products/human-alpha-fetoprotein-afp-antibody-189506_mab1369
- ALB R&D Systems AF3329 1:500; https://www.rndsystems.com/products/human-mouse-serum-albumin-antibody_af3329
- NKX6-2 abcam ab58708 1:100; https://www.abcam.com/nkx62gtx-antibody-ab58708.html
- ROBO2 Cell Signaling Technology 45568S 1:100l; https://www.cellsignal.com/products/primary-antibodies/robo2-e4m6d-rabbit-mab/45568
- GP2 MBL INTERNATIONAL D277-5 3G7-H9 1:100; https://www.mblintl.com/products/d277-3/
- INS Dako a0564 1:1000; https://antibodyregistry.org/search.php?q=AB_10013624
- H3K4me1 abcam ab8895 1:50; https://www.abcam.com/histone-h3-mono-methyl-k4-antibody-chip-grade-ab8895.html
- H3K27ac abcam ab4729 1:50; https://www.abcam.com/histone-h3-mono-methyl-k4-antibody-chip-grade-ab8895.html
- FOXA1 abcam ab170933 EPR10881 1:50; https://www.abcam.com/foxa1-antibody-epr10881-chip-grade-ab170933.html
- CD184-PECy7 BDBiosciences 560669 12G5 (RUO) 1:100; https://www.bdbiosciences.com/en-us/products/reagents/flow-cytometry-reagents/research-reagents/single-color-antibodies-ruo/pe-cy-7-mouse-anti-human-cd184.560669
- CD117-APC BDBiosciences 561118 YB5.B8 (RUO) 1:100; https://www.bdbiosciences.com/en-us/products/reagents/flow-cytometry-reagents/research-reagents/single-color-antibodies-ruo/apc-mouse-anti-human-cd117.561118
- Alexa fluor 488 anti-goat Thermo Fisher A11055 1:800; https://www.thermofisher.com/antibody/product/Donkey-anti-Goat-IgG-H-

L-Cross-Adsorbed-Secondary-Antibody-Polyclonal/A-11055
- Alexa fluor 568 anti-goat Thermo Fisher A11057 1:800; https://www.thermofisher.com/antibody/product/Donkey-anti-Goat-IgG-H-L-Cross-Adsorbed-Secondary-Antibody-Polyclonal/A-11057
- Alexa fluor 647 anti-goat Thermo Fisher A21447 1:800; https://www.thermofisher.com/antibody/product/Donkey-anti-Goat-IgG-H-L-Cross-Adsorbed-Secondary-Antibody-Polyclonal/A-21447
- Alexa fluor 488 anti-mouse Thermo Fisher A21202 1:800; https://www.thermofisher.com/antibody/product/Donkey-anti-Mouse-IgG-H-L-Highly-Cross-Adsorbed-Secondary-Antibody-Polyclonal/A-21202
- Alexa fluor 568 anti-mouse Thermo Fisher A10037 1:800; https://www.thermofisher.com/antibody/product/Donkey-anti-Mouse-IgG-H-L-Highly-Cross-Adsorbed-Secondary-Antibody-Polyclonal/A10037
- Alexa fluor 488 anti-rabbit Thermo Fisher A32790 1:800; https://www.thermofisher.com/antibody/product/Donkey-anti-Rabbit-IgG-H-L-Highly-Cross-Adsorbed-Secondary-Antibody-Polyclonal/A32790
- Alexa fluor 568 anti-rabbit Thermo Fisher A10042 1:800; https://www.thermofisher.com/antibody/product/Donkey-anti-Rabbit-IgG-H-L-Highly-Cross-Adsorbed-Secondary-Antibody-Polyclonal/A10042
- Alexa fluor 647 anti-rabbit Thermo Fisher A32795 1:500l; https://www.thermofisher.com/antibody/product/Donkey-anti-Rabbit-IgG-H-L-Highly-Cross-Adsorbed-Secondary-Antibody-Polyclonal/A32795

The above antibodies are also used routinely in our laboratory and have been tested on numerous protocols and shown to be specific.

Additional validation was done by the use of negative control and control tissue samples.

Eukaryotic cell lines

Policy information about cell lines

Cell line source(s)	Human embryonic stem cell lines used in this study were published:<br>1. H9 WT hESC (WA09) was obtained from WiCell (WiCell Madison, WI)<br>2. HUES4 WT hESC was obtained from D.A. Melton, Howard Hughes Medical Institute (Harvard University, Cambridge, MA).<br>3. PDXeG clone 170-3 hESC was from Henrik Semb. (Ameri et al Cell Reports, 2017 vo.19 (1) pp.36-49)
Authentication	The cell lines were karyotyped and tested for differentiation competence.
Mycoplasma contamination	The cell line has been tested negative for mycoplasma contamination
Commonly misidentified lines<br>(See ICLAC register)	No cell lines used in this study were found in the database of commonly misidentified cell lines that is maintained by ICLAC and NCBI Biosample.

Flow Cytometry

Plots

Confirm that:

☒ The axis labels state the marker and fluorochrome used (e.g. CD4-FITC).

☒ The axis scales are clearly visible. Include numbers along axes only for bottom left plot of group (a 'group' is an analysis of identical markers).

☒ All plots are contour plots with outliers or pseudocolor plots.

☒ A numerical value for number of cells or percentage (with statistics) is provided.

Methodology

Sample preparation	Human ESC, ADE, VFG, and pancreatic spheroids were dissociated with Trypsin 0.25%/EDTA, and then resuspended in FACS buffer (PBS/FBS) to stop dissociation.
Instrument	Sony SH800 and BD LSR Fortessa.
Software	Cells were sorted on a 4 laser (488nm, 561nm, 638nm and 405nm) SONY SH800 and acquisition was done using Sony SH800 software version 1.8. The acquisition of BD LSR Fortessa (488nm, 405nm, 355nm, 640nm, 561nm) was done using BD FACSDiva software version 8. Data was analyzed using FCS Express 6.
Cell population abundance	Human ESC culture contained more than 80% live cells. Differentiating ADE cells contained around 60% live cells which were CD184-CD117 double positive. In expanding VFG cell cultures, more than 80% of live cells were CD184-CD117 double positive. Pancreatic spheroids generated from VFG (with PDX1-eGFP reporter) contained more than 80% of GFP positive cells.
Gating strategy	Cells were first gated based on similar light scatter properties, doublets gated out using the width parameter on forward scatter, and then live cells gated based on DAPI. For conjugated Ab cell surface marker staining, negative (isotype control) cells were used as gating parameter.

☒ Tick this box to confirm that a figure exemplifying the gating strategy is provided in the Supplementary Information.
